# Human recreation affects spatio-temporal habitat use patterns in red deer (*Cervus elaphus*)

**DOI:** 10.1371/journal.pone.0175134

**Published:** 2017-05-03

**Authors:** Joy Coppes, Friedrich Burghardt, Robert Hagen, Rudi Suchant, Veronika Braunisch

**Affiliations:** 1 Forest Research Institute of Baden-Württemberg (FVA), Freiburg, Germany; 2 Black Forest National Park, Seebach, Germany; 3 Conservation Biology, Institute of Ecology and Evolution, University of Bern, Bern, Switzerland; Université de Sherbrooke, CANADA

## Abstract

The rapid spread and diversification of outdoor recreation can impact on wildlife in various ways, often leading to the avoidance of disturbed habitats. To mitigate human-wildlife conflicts, spatial zonation schemes can be implemented to separate human activities from key wildlife habitats, e.g., by designating undisturbed wildlife refuges or areas with some level of restriction to human recreation and land use. However, mitigation practice rarely considers temporal differences in human-wildlife interactions. We used GPS telemetry data from 15 red deer to study the seasonal (winter vs. summer) and diurnal (day vs. night) variation in recreation effects on habitat use in a study region in south-western Germany where a spatial zonation scheme has been established. Our study aimed to determine if recreation infrastructure and spatial zonation affected red deer habitat use and whether these effects varied daily or seasonally. Recreation infrastructure did not affect home range selection in the study area, but strongly determined habitat use within the home range. The spatial zonation scheme was reflected in both of these two levels of habitat selection, with refuges and core areas being more frequently used than the border zones. Habitat use differed significantly between day and night in both seasons. Both summer and winter recreation trails, and nearby foraging habitats, were avoided during day, whereas a positive association was found during night. We conclude that human recreation has an effect on red deer habitat use, and when designing mitigation measures daily and seasonal variation in human-wildlife interactions should be taken into account. We advocate using spatial zonation in conjunction with temporal restrictions (i.e., banning nocturnal recreation activities) and the creation of suitable foraging habitats away from recreation trails.

## Introduction

An increasing number of people are practicing nature-based tourism, with outdoor recreation activities generating pressure on the ecosystems in which they take place [1, 2]. An important factor of how outdoor recreation affects ecosystems is the disturbance of wildlife by human recreation [3, 4], defined here as any effect on wildlife which is incurred by the presence of recreationists or infrastructure related to recreational activities, irrespective of possible—but mostly unknown—fitness consequences [5, 6]. Free-living animals often react to human presence in a similar way than to the presence of natural predators [7, 8]. This reaction can have a variety of facets [9, 10], ranging from physiological stress responses [11–14] to behavioural changes [8, 15] or a reduction in reproductive success [8]. Human disturbance might trigger short-term behavioural reactions (i.e. flushing or fleeing) [1, 16, 17] as well as long-term responses such as avoiding frequently disturbed areas [18, 19], e.g. recreational infrastructures such as hiking or skiing trails that are regularly used by humans [20]. Both types of reaction can involve direct energetic costs for the animal (e.g. due to fleeing or reduced food intake) which can affect fitness [14, 21, 22], and may even outweigh the effects of habitat conditions and natural predators [23].

In addition, the reactions of wildlife triggered by human recreational activities can cause conflicts with other forms of human land use, such as transportation, agriculture or forestry. For example, fleeing animals can trigger vehicle collisions [24], and foraging animals relocating to less disturbed areas might cause damage to crop or tree regeneration [25, 26].

To mitigate both the negative effects of human recreation on wildlife and the resulting conflicts with land use, spatial zonation schemes have become an important tool in wildlife management [27]. These schemes separate human activities from key wildlife habitats by designating undisturbed wildlife refuges and areas with different levels of restriction to human recreation, sometimes combined with habitat management or hunting regulations. The design of zonation schemes often takes spatial patterns of human-wildlife interactions into account but rarely considers temporal interactions, e.g. variation in diurnal and seasonal overlaps between habitat requirements and recreation activities. Using the red deer (*Cervus elaphus*) as an example organism, we investigated the temporal variation of its habitat use in relation to human recreation infrastructure and zones with different intensities of human disturbance, as established by a zonation scheme.

As one of the largest free ranging herbivores, and widely distributed across the globe [28, 29], the red deer is one of the focal species of wildlife management in Central Europe [30, 31]. Red deer are attractive to observe and are therefore highly valued for nature-based tourism [30] and also as a game species [28, 29]. They are considered an important vector species for seeds [32, 33] and invertebrates [34] and an essential prey for carnivores (e.g. wolf) [35]. With its browsing behaviour it can affect the vegetation structure [36–38] and there is some evidence for impacts on plant species richness [39]. At the same time however, deer browsing and bark stripping causes conflicts with forestry management [31, 40, 41]. In addition, thousands of individuals are injured or die in vehicle collisions every year, causing considerable property damage and fatal human injuries [42, 43]. The major objective of the red deer management in Europe is therefore to minimize the economic damage related to forestry and animal vehicle collisions while maximizing the economic benefits related to ecosystem services and hunting [44–46].

Outdoor recreation has been widely neglected within the management of free ranging ungulates [1], although red deer have been shown to be influenced by human recreationists [20, 26, 47, 48]. Direct reactions to disturbance include instant flight, relocation to areas with dense vegetation cover [26, 49] as well as a temporal abandonment of the disturbed area (i.e. for several hours or days) [26]. Sibbald et al. [20] found red deer avoided hiking trails, with larger distance to the trail kept during times of high human use compared to times of little use. In areas with high recreation pressure, red deer have been shown to increase their vigilance behaviour which might lead to a decrease in food uptake [47]. Animals can also adjust their habitat use between hunting season and non-hunting season [50], which indicates their behavioural plasticity. However, even though there are several examples where north American elk (*Cervus elaphus canadensis*) have become habituated to human presence [51] and even use settlements as habitat [52], this phenomenon is not known from free-ranging European red deer [53, 54]. Human disturbance may therefore cause red deer to temporally or permanently abandon optimal habitat and forage in sub-optimal habitats [48]. Increased energy requirements caused by fleeing, in conjunction with seeking cover [26] could result in damage to forestry e.g. through bark-stripping in young, dense stands offering visual protection.

The most widely applied method of red deer management involves hunting, to regulate the population and to gain trophies (antlers) and meat [28, 29]. Furthermore in many areas red deer are provided with supplementary food during winter to reduce bark-stripping or because of animal welfare reasons [55]. In the last decades, wildlife refuges have increasingly been designated, with the primary aim to reduce disturbance of deer by recreationists, land use management and hunters [25, 56, 57]. However, it has also been suggested that well-placed refuges may help reduce human-wildlife conflicts [58] and contribute to decreasing damage to forestry by reducing the browsing pressure on the surrounding forest stands [25]. To serve this purpose, refuge systems have been extended to spatial zonation schemes that regulate recreational activities but also hunting and forest management [27]. However, management schemes aiming at furthering the coexistence of humans and wildlife must also consider temporal dimensions of human-wildlife interactions [18, 59]. Given the seasonal and diurnal differences in recreation activities and the behavioural plasticity of red deer, we expect that the spatial pattern of habitat use in relation to human recreation infrastructure varies considerably between seasons and between day and nighttime, which might also modify the relative importance (i.e. intensity of use) of the zones of a static spatial zonation. To test this, we studied the habitat use of free roaming red deer comparing daytime and nighttime activity in two different seasons using GPS-telemetry. The study was conducted in a red deer management area in south-western Germany in which a spatial zonation scheme had been established, defining red deer refuges (without human recreation), a core zone with limited recreational use and a border zone with unrestricted recreation. The goals of our study were to determine if linear recreation infrastructure (i.e. hiking, biking and skiing trails) and the zonation scheme affected red deer habitat use and whether these effects varied daily or seasonally. From the results we derive recommendations for mitigating impacts of human recreation on ungulates in human-dominated landscapes.

## Methods

### Ethics statement

Red deer capturing and tagging was carried out under the permit (No. 787.524) issued by the ethical committee of the Regional Council of Freiburg, Baden-Württemberg (Regierungspräsidium Freiburg, Baden-Württemberg). The ethical committee specifically approved this study. GPS collars were attached under anesthesia (125 mg Xylazine + 100 mg Ketamine /ml).

### Study area

**Fig 1:**
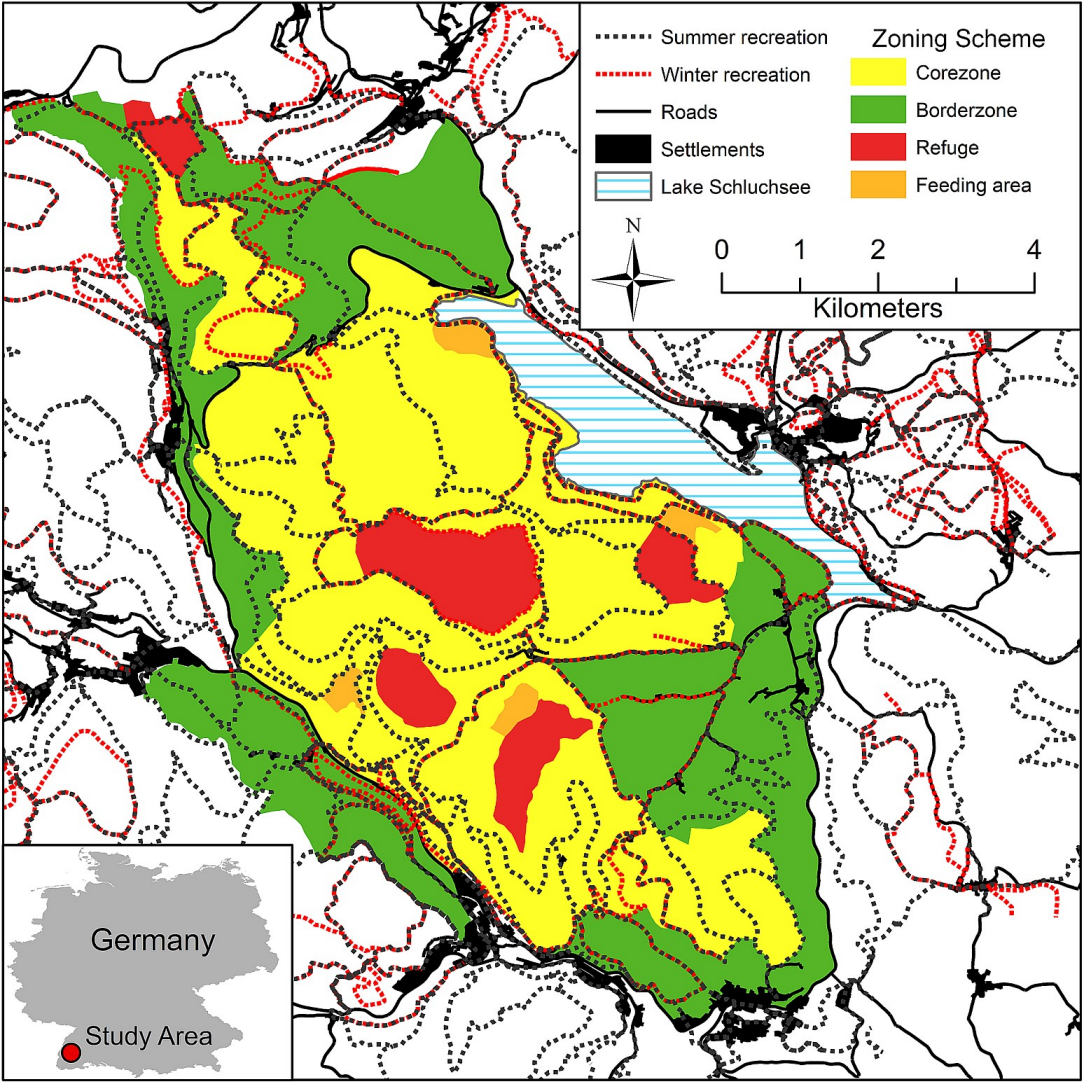
Study area. The study area in south-western Germany, with recreation infrastructure (summer/winter) and spatial zonation defining border, core and refuge zones, with different implications for red deer management.

The study was conducted in the Southern Black Forest, Baden-Württemberg, south-western Germany (Fig 1). In the state of Baden-Württemberg it is official policy to try to keep red deer in five specially designated areas, which are mainly state owned, to avoid conflicts with private forest owners and farmers. Red deer leaving the management areas are shot at sight. The Southern Black Forest red deer management area has a total surface of 17500 ha; our study was performed in the central part of 5984 ha, located at elevations between 800 and 1300m above sea level (a.s.l.). Most of the study area (77%) consists of intensively managed forest (for timber production) dominated by Norway spruce (*Picea abies*), European silver fir (*Abies alba*) and common beech (*Fagus sylvatica*) [60]. Extensively managed meadows prevail in the non-forested areas.

The study area is located between two major tourist attractions Lake Schluchsee and the Feldberg Mountain and is intensively and increasingly used for recreation all year round. This is reflected in a 24% increase of tourist visits to the region between 2004 and 2014 [61]. In the study area, a dense network of recreation trails has been established: in summer, a total of 162 km (2.71 km/km^2^) of paths are accessible, mainly for hiking and biking. During winter, trails for hiking (48 km; 0.8km/km^2^) and cross-country skiing (39 km; 0.65km/km^2^) are prepared, but there are also off-trail recreational activities (i.e. snowshoeing and back-country skiing) [62]. To assess the temporal patterns of human recreationists in the areas, automatic visitor counts were performed on hiking and skiing trails one year after the data collection for the telemetry study. Infrared trail counters (TRAFx), were placed along three designated hiking trails and three cross country skiing trails within the study area from 17.2.2010 to 14.4.2010, showing a peak of recreation activities at noon, with an average number of six persons per hour recorded on hiking trails and two per hour on skiing trails (S2 Fig).

During the duration of our study (2007–2009), 600–700 free-roaming red deer were estimated to be present in winter within the total red deer management area (Forest Research Institute of Baden-Württemberg FVA, unpublished), which corresponded to a density of 3.43–4.00 individuals per km^2^. Other ungulates present are roe deer (*Capreolus capreolus*), wild boar (*Sus scrofa*) and sika deer (*Cervus nippon*) (FVA, unpublished). Predators include red fox (*Vulpes vulpes*) and pine marten (*Martes martes*), but with lynx (*Lynx lynx*) and grey wolf (*Canis lupus*) absent, adult red deer have no natural predators in the area (FVA, unpublished).

### Zoning scheme

Beginning in 2003, a spatial zonation scheme was developed and implemented using a joint participative process, which included wildlife biologists, foresters, hunters and landowners. It was officially approved in 2008 by the local communities. The scheme includes different zones with regulations concerning not only recreational use, but also hunting, forestry and red deer habitat management (Fig 1, Table 1) [56]: A border zone—where no restrictions for recreation apply—surrounds a core zone where recreation is restricted to designated trails. Embedded within the core zone, refuge areas for red deer have been designated, where recreational use is totally banned. During winter the deer are fed at four feeding stations to minimize seasonal migration and thus reduce deer-vehicle collisions, but the animals are not fenced during any time of the year [56].

**Table 1:** Spatial zonation of the study area with management conditions.

Zone	Recreation	Hunting	Forestry	Habitat improvement
Border zone	No restrictions	No hunting between 31st December and 1^st^ of August	Local adaptations to prevent damage where necessary	Locally: measures to increase natural food supply
Core zone	Access only on marked trails	Only August-November, only interval hunting	Browsing damage and additional effort for damage prevention accepted	Increase of natural food supply
Refuges	Access prohibited	Only three consecutive weeks per year (outside reproduction season)	As in core zone; and no forestry during the reproductive season	Increase of natural food supply and cover
Feeding stations	Access prohibited during winter	No hunting	As in core zone; and no forestry during feeding times	Promotion of cover and reduction of visibility from marked trails

In the study area the hunting times are more restricted compared to the official state hunting regulations. In the border zone, hunting is only allowed from the 1^st^ of August until 31^st^ of December (i.e banned in the summer hunting season between May and July). In the core zone hunting activities are additionally banned in December and restricted to interval hunting (i.e. short hunting intervals followed by several days without hunting with the goal to reduce disturbance). In the refuge areas hunting is restricted to driven hunts in three consecutive weeks in October. The aim of the hunting regime in the area is to limit the red deer population size (winter) to an overall number of 400 individuals (2.29 individuals per km^2^).

In all zones, forestry is directed towards creating small openings during timber harvesting, to increase natural food resources for the deer. In the border zone, moderate protection measures, such as small scale fencing, can be implemented to avoid damage to forestry caused by deer. In the core zone, damage caused by deer to forestry is accepted. In the refuges and feeding stations no forestry measures (i.e. timber harvesting) are performed during the fawning season (i.e. May and June) and feeding times (i.e. snow conditions). The main goals of this zonation scheme are decreasing damage to forestry across the whole area by allowing the deer to retreat to undisturbed areas with sufficient food in summer and additional feeding in winter, while at the same time creating possibilities for human recreation which includes the possibility to observe and experience red deer [56].

### Red deer data

Our analysis was based on telemetry locations of 15 red deer (5 males and 10 females, all age classes, S1 Table) captured and surveyed between 2007 and 2009. Individuals were equipped with a GPS-collar (Vectronic Aerospace, Berlin, Germany; serial number 2000er, 3000er and 6000er) and located every 2 hours. The tracking period of individual animals ranged between 5 and 34 months (S1 Table), depending on the functional duration of the GPS collars and due to individual fatality events. We retained only locations if a minimum of 4 satellites were available and the DOP (dilution of precision) value was smaller than 10 (corresponding to an estimated maximum location error of about 40m [63]), resulting in 80% of the locations for further analysis. To model temporal differences in habitat use, each sample was allocated to a season (summer, winter) and a time of day (day, night). Since seasonal differences in habitat use patterns were assumed to be related to prevailing weather conditions rather than being determined by a predefined time period, seasons were defined using standard indicators of weather conditions: The “summer” season started with the flowering of dandelion (*Taraxacum officinale*) (18, 22 and 30 April in 2007, 2008 and 2009, respectively), as measured at the phenology reference station Bernau, 920 m.a.s.l., and ended with the start of the rutting season (15 September, all years). The “winter”-sample contained all locations taken between first of November and the beginning of the summer season in the following year, including only days where a continuous snow-layer was recorded at the nearby weather station (St. Blasien-Menzenschwand, 885 m.a.s.l.). Locations taken outside the defined seasons were discarded.

Among the retained locations, we distinguished between day and night. Day was defined as the time between sunrise and sunset, and night covered the time between the end and the start of the nautical twilight. Due to failing fixes or fixes with too high DOP (i.e. low precision), the number of locations per day and time period varied greatly within and between individuals. To avoid an unbalanced sample, (i.e. some time periods being overrepresented by data showing high spatial and temporal autocorrelation) we adopted a conservative approach, randomly selecting only one location per time period and day for every individual.

Of the resulting 24259 locations which were retained for further analysis (S1 Table), 7384 locations pertained to summer, and 16875 to the winter season. The number of locations per individual varied between 244 and 3136 (S1 Table).

### Environmental variables

We distinguished three groups of environmental predictors, pertaining to land cover and topography, vegetation structure and human presence (Table 2).

**Table 2:** Predictor variables included in the models.

Predictor type	Variable Name	Description (unit)	Min-Max	Type
Landscape and	DHM	Altitude (m a.s.l.)	762–1314	continuous
topography	SLOPE	Slope (degree)	0–44	continuous
	NORTHING	Northness (cosine aspect)	-1–1	continuous
	EASTING	Eastness (sine aspect)	-1–1	continuous
	WATER	Proximity to lakes, rivers and creeks (km)	0.005–0.704	continuous
	GREENL	Proximity to greenland (i.e. meadows/ grassland) (km)	0–1.343	continuous
	FOREST_250	Forest cover within a 250m radius (%)	0–100	continuous
Vegetation	CANOPY_TYPE	Type of canopy trees		categorical
		CAN_NO = No forest (reference category)		
		CAN_CON = Coniferous >95%		
		CAN_CONMIX = Conifer dominated mixed (conifers >50%)		
		CAN_DEC_MIX = Deciduous dominated mixed (deciduous >50%)		
		CAN_DEC = Deciduous >95%		
	CANOPY_COV	Canopy cover (%)	0–100	continuous
	SUCCESSION	Successional stage		categorical
		SUC_OPEN = Open (reference category)		
		SUC_REGTHICK = Regeneration & Thicket		
		SUC_POLE = Pole stage		
		SUC_TREE = Tree stage		
		SUC_OLD = Old forest		
	UNDER_TYPE	Type of understory trees		categorical
		UNDER_NON = No understorey (reference category)		
		UNDER_CON = Coniferous >95%		
		UNDER_DEC = Deciduous >95%		
		UNDER_DECMIX = Deciduous dominated mixed (deciduous >50%)		
		UNDER_CONMIX = Conifer dominated mixed (conifers >50%)		
	UNDER_COV	Cover of understory (%)	0–90	continuous
	BILBERRY	Bilberry cover (%)	0–90	continuous
	HERB_GRAS	Cover of herbs and grass (%)	0–100	continuous
	PROTECTION_S/W	Protection from visibility in summer/winter (%)	0–75	continuous
Human presence	TOURI_S/W	Proximity to summer tourism infrastructure in summer/winter (km)	S: 0–0.752	continuous
			W: 0–1.824	
	TOURI_DENS_S/W	Density of summer/winter tourism infrastructure within 250m	S: 0–129	continuous
		(m/ha)	W: 0–108	
	ROAD	Proximity to roads (km)	0.006–2.321	continuous
	SETTLE	Proximity to settlements (km)	0–3.105	continuous
	FEED	Proximity to feeding stations (km)	0–6.300	continuous
	HUNT	Proximity to hunter hides (km)	0–3.918	continuous
	MGT	Different area-types of the red-deer management scheme (Table 1)		categorical
		MGT_BORDER = Border zone (reference category)		
		MGT_CORE = Core area		
		MGT_REFUGE = Refuge area		

Topographic variables (altitude, slope and exposition) were calculated from the digital elevation model (DEM). Land cover characteristics (waterbodies, meadows, forest) were adopted from the Official Topographic and Cartographic Information System of Germany (ATKIS, www.atkis.de).

Vegetation was mapped in the field: forest stand type, canopy cover, tree-species mixture, successional stage, understory composition, cover of herbs and grass as well as bilberry cover (*Vaccinium myrtillus*) was recorded for forest stand units, which represent homogenously structured patches with a mean size of 3.40 ha (min: 0.20, max: 48.20). The variable “visual protection” in summer and winter was recorded in a location where understory conditions were considered representative for the respective forest stand. Using a “chessboard” (100x100cm) with a black and white grid (i.e. 100 10 x10cm squares), placed upright at a distance of 30 m in all four cardinal directions from the observer, the amount of visual protection was then derived from the number of squares that were hidden by the vegetation. Covering an area of 10% of the average stand size, this measurement provides a rough estimation of the possibility for red deer to hide. As vegetation mapping was done in summer, protection in winter was estimated based on the understory type and density, i.e. subtracting the cover provided by broadleaved trees and bushes.

Human infrastructure (roads, settlements) was accessed from the Official Topographic and Cartographic Information System of Germany (ATKIS, www.atkis.de). In addition, we mapped tourism infrastructure in summer (hiking trails, mountain bike routes) and winter (cross country skiing and snowshoe trails, winter hiking paths), the location of the red-deer feeding stations in winter and the different zones of the zoning scheme. For all predictors we prepared raster maps with a 10 x 10 m resolution. To account for potential radio tracking errors, we performed a circular moving window analysis with a radius of 40m (corresponding to the maximum location error), assigning to the focal cell the mean value or, in case of categorical variables, the category that was most frequently present within the window. Variable maps were processed in ArcGIS 9.3 (ESRI 2009).

### Statistical approach

To analyze habitat use we adopted a ‘used versus available’ design at two spatial habitat scales, comparing the presence data with two sets of random locations: First, to determine the factors influencing home range selection within the study area (second order habitat selection [64]), the presence locations of each individual were contrasted against the same number of random locations generated throughout the study area. Second, to analyze habitat selection within the home range (third order habitat selection [64]), we generated a second random sample selected from the individuals’ seasonal home ranges. Home ranges were calculated for each year and season separately, using the full data set (i.e. all available locations of the individual for the season) and the 100% minimum convex polygon (MCP) method. Habitat use was analyzed using Generalized Linear Mixed Effects Models (GLMM, R-package: lme4 [65]) with a logit link and binomial error structure, including the individual as a random factor. First, starting with the initial set of variables (Table 2), we identified pairs of strongly correlated variables (Spearmans’ R_s_ > |0.5|), discarding the variable that explained less within a univariate model. Multivariate models with all possible combinations of the remaining variables were then fitted using the dredge function (R-package MuMin, [66]) in order to find the most parsimonious model according to Akaikes Information Criterion (AIC) [67]. Model averaging was applied if several “best models” did not differ significantly (Δ AIC <2). For each season we fitted three models describing (1) home range selection within the study area (day and night pooled), as well as habitat use within the home range during (2) day and (3) night. In addition, we tested for differences in habitat use between day and night, identifying the environmental predictors that significantly discriminated the individual’s locations taken at the two different time periods. To assess multicollinearity in the final models (i. e. whether linear combinations of the fixed effects were correlated), we calculated the variance inflation factor (VIF) for all models, using the corvif function in the R-package AED [68]. For continuous variables we accepted an VIF of less than 10 [69], for factor variables the VIF was corrected for the number of degrees of freedom (VIF^(1/2df)) [68]. The importance of individual variables was evaluated by fitting the final models while leaving out the respective variable. The change in AIC (ΔAIC) compared to the final model was then used as an indicator of the variable’s relative contribution to the final model. In addition, for every independent variable we calculated the odds-ratio and its 95% confidence interval using the Wald chi-square test [70] to approximate its effect on the dependent variable [70]. Model performance was evaluated using the area under the receiver operating characteristic (ROC) curve (AUC, R-package: AICcmodavg [71]). All statistical analyses were performed using the software R (R Version 2.15.1, www.rproject.org).

## Results

### Home range location

According to the classification of Hosmer and Lemeshow [72] our models performed well in explaining home range selection within the study area during both summer (AUC: 0.766 ± 0.003) and winter (0.919 ± 0.002). Home range selection was explained by variables describing landscape, vegetation structure and human presence (Table 3). According to the ΔAIC (Table 3), the zonation scheme was the strongest predictor for home range selection in both seasons: in summer the refuge areas were selected over the core area and the border zone, which served as a reference category (Table 3A), whereas winter home range selection was mainly located close to the feeding stations. The effect of human infrastructure differed between seasons: whereas in summer human settlements were avoided, winter home ranges were selected in closer vicinity to settlements and roads than expected from a random selection. The proximity and density of recreation infrastructure had no significant effect on home range selection. During the summer months deer home ranges were located in forest areas with a high proportion of openings and thickets rich in herbs and grasses whereas in winter older stands (pole and tree stage, and old forest) and south-eastern facing slopes were selected (cf. Table 3 for home range selection in the study area and S3–S7 Tables for further information regarding the model selection, VIF and odds ratios).

**Table 3:** Selection of home range in study area.

		(a) Summer (AUC 0.766 +- 0.003)				(b) Winter (AUC 0.919 +- 0.002)			
		SD (Individual): 0.041 AIC: 24334				SD (Individual): 0.059 AIC: 8488			
Type	Variable	Estimate	SE	Sign.	ΔAIC	Estimate	SE	Sign	ΔAIC
	INTERCEPT	-1.844	0.103	***		2.977	0.185	***	
Vegetation	SUC_REGTHICK	0.449	0.277		71	2.209	0.245	***	227
	SUC_POLE	-0.083	0.270			1.026	0.210	***	
	SUC_TREE	0.031	0.268			1.771	0.190	***	
	SUC_OLD	-0.200	0.272			1.898	0.198	***	
	PROTECT_S/W	<0.001	0.001		2	-0.010	0.002	***	31
	BILBERRY	-0.008	0.002	***	23				
	CAN_CON	-0.981	0.268	***	45				
	CAN_DEC	0.404	0.413						
	CAN_CONMIX	-0.874	0.269	**					
	CAN_DECMIX	-0.761	0.276	*					
	HERB_GRAS	0.018	0.001	***	498				
	CANOPY_COVER					-0.011	0.003	***	16
Landscape	WATER	1.146	0.171	***	48	-0.856	0.286	***	9
	FOREST250	0.536	0.106	***	32				
	SLOPE	-0.004	0.003		4				
	NORTHING					-0.270	0.046	***	36
	EASTING					-1.054	0.054	***	376
Human	MGT_CORE	1.930	0.064	***	1538	-0.498	0.090	***	133
	MGT_REFUGE	2.723	0.077	***		0.529	0.125	*	
	FEED					2.055	0.057	***	2098
	HUNT	1.762	0.051	***	1527	1.896	0.092	***	544
	SETTLE	-0.347	0.028	***	137	0.368	0.068	***	67
	TOURI_S/W	0.147	0.132		2				
	ROAD					0.684	0.102	***	31

Variables determining the home range selection of red deer within the study area in (a) summer and (b) winter. For all variables positive estimates indicate preference, negative estimates indicate avoidance. For predictor names see Table 2. Significance levels are indicated with: * p ≤ 0.05, ** p ≤ 0.01, and *** p ≤ 0.001. Relative variable importance is indicated by ΔAIC, which is the difference in AIC of a model discarding the respective variable compared to the full model.

### Habitat use within the home range

**Fig 2:**
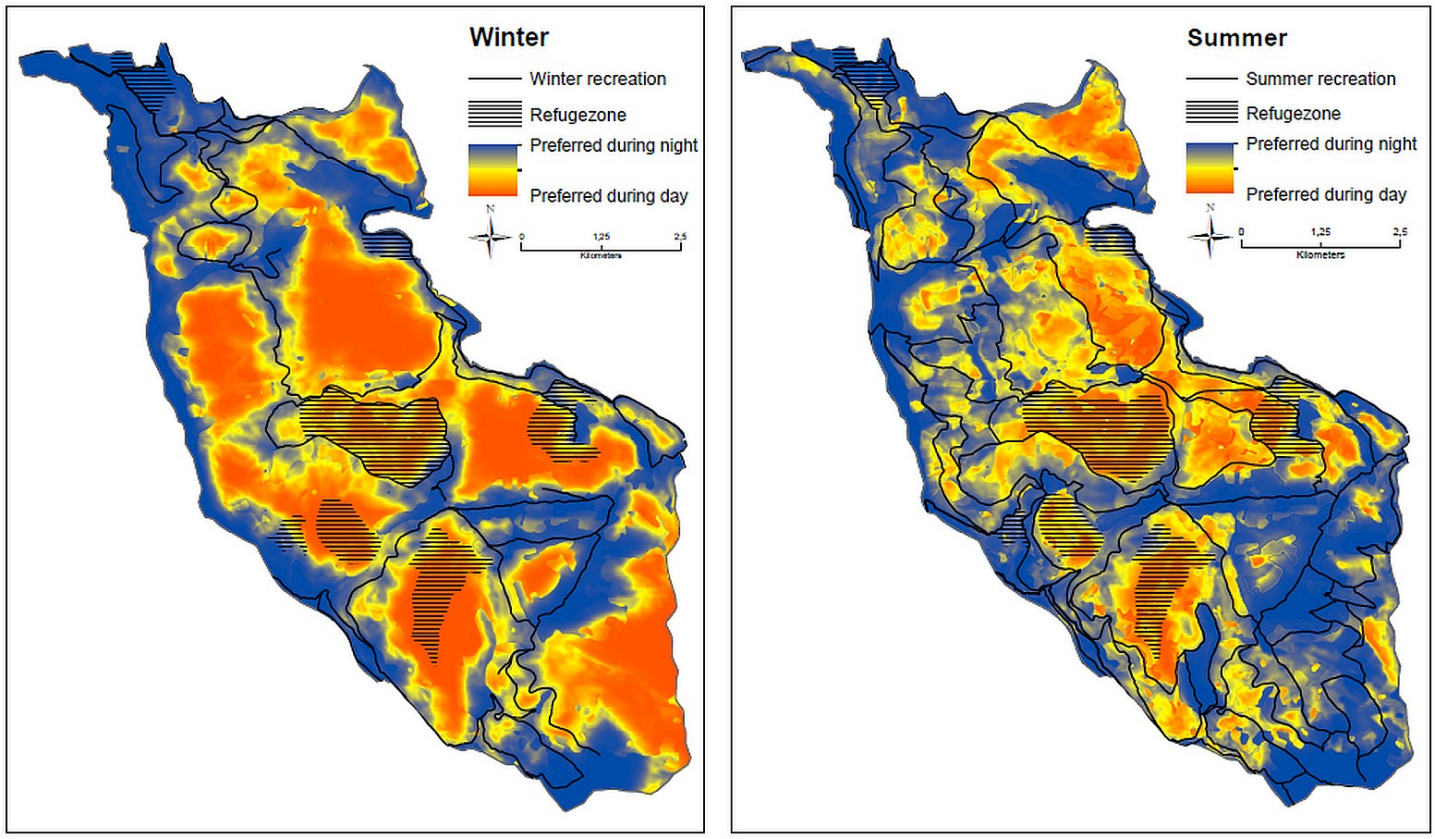
Differences in red deer habitat use between day and night during the winter (left) and summer (right). Red areas indicate zones that are more often used during daytime, while blue areas are more frequented during nighttime. Yellow areas are similarly used during day or night. The hatched areas indicate the location of the refuge zones. The probability of red deer presence for both seasons and times of the day are shown in S1 Fig.

Habitat use within the home range during summer and winter was explained by vegetation, land use and human presence (Table 4). The zoning scheme also ranked among the most important predictors, with refuges and core areas being selected over the border zones in summer (Table 4, S2 Table, available online in Supporting Information). In winter red deer aggregated at the feeding sites during the day, whereas the refuges were predominantly selected during the night (Table 4). However, habitat use differed significantly between the day and nighttime, particularly with regard to the variables related to human presence (Fig 2, Tables 4 and 5). Both in summer and winter recreation trails were avoided during day, whereas a positive association could be found during night (Table 4). In addition, red deer selected areas with shallower slopes and in greater vicinity to water during night. In the summer season, red deer visited bilberry patches during night that were avoided during daytime, whereas in the winter season, they stayed more frequently in the vicinity of roads during nights compared to daytime (Table 5).

**Table 4:** Habitat use within the home range in summer and winter, day and night.

Summer	Day (AUC: 0.684 +- 0.005)					Night (AUC: 0.810 +- 0.005)			
	SD (Individual): 0.329 AIC: 12040					SD (Individual): 0.450 AIC: 7317			
Type	Variable	Estimate	SE	Sign.	ΔAIC	Estimate	SE	Sign.	ΔAIC
	INTERCEPT	-4.675	0.226	***		2.190	0.188	***	
Vegetation	CANOPY_COVER	-0.013	0.002	***	37	-0.036	0.002	***	243
	**SUC_REGTHICK**	**2.412**	**0.224**	***	**352**	**0.498**	**0.466**		**19**
	**SUC_POLE**	**1.351**	**0.204**	***		**0.743**	**0.437**	*	
	**SUC_TREE**	**0.653**	**0.199**	**		**0.732**	**0.433**	.	
	**SUC_OLD**	**0.982**	**0.202**	***		**0.203**	**0.436**		
	**BILBERRY**	**-0.025**	**0.003**	***	**59**	**0.022**	**0.003**	***	**48**
	PROTECT_S	-0.004	0.001	**	6				
	UNDER_CON	0.731	0.100	***	143				
	UNDER_DEC	1.850	0.245	***					
	UNDER_CONMIX	0.674	0.079	***					
	UNDER_DECMIX	0.148	0.088	.					
	**CAN_CON**					**-0.760**	**0.433**	.	**26**
	**CAN_DEC**					**0.405**	**0.617**		
	**CAN_CONMIX**					**-0.678**	**0.434**		
	**CAN_DECMIX**					**-1.330**	**0.468**	**	
Landscape	**WATER**	**-2.487**	**0.257**	***	**160**	**1.175**	**0.361**	***	**11**
	**EASTING**	**0.083**	**0.042**	*	**2**	**-0.409**	**0.055**	***	**68**
	**SLOPE**	**0.064**	**0.004**	***	**213**	**-0.042**	**0.006**	***	**51**
	NORTHING	-0.279	0.035	***	60				
	**FOREST250**	**1.583**	**0.224**	***	**51**				
Human	MGT_CORE	1.282	0.158	***	73	1.371	0.136	***	116
	MGT_REFUGE	1.249	0.165	***		1.739	0.163	***	
	**TOURI_S**	**-1.616**	**0.189**	***	**157**	**0.865**	**0.242**	*	**5**
	**HUNT**	**1.159**	**0.118**	***	**172**				
	SETTLE					0.254	0.064	***	33
	ROAD					0.503	0.079	***	27
Winter	Day (AUC: 0.849 +- 0.006)					Night (AUC: 0.880 +- 0.005)			
	SD (Individual): 0.378 AIC: 4067					SD (Individual): 0.300 AIC: 3405			
Type	Variable	Estimate	SE	Sign.	ΔAIC	Estimate	SE	Sign.	ΔAIC
	INTERCEPT	0.492	0.350			1.992	0.250	***	
Vegetation	CANOPY_COV	-0.012	0.004	**	8				
	**CAN_CON**	**3.761**	**0.379**	***	126				
	**CAN_DEC**	**2.887**	**1.377**	*					
	**CAN_CONMIX**	**3.410**	**0.387**	***					
	**CAN_DECMIX**	**3.720**	**0.454**	***					
	SUC_REGTHICK					-1.130	0.357	***	281
	SUC_POLE					-1.173	0.215		
	SUC_TREE					0.950	0.158	***	
	SUC_OLD					1.311	0.219	***	
	PROTECT_W					-0.001	0.004	***	11
Landscape	**NORTHING**	**0.241**	**0.074**	**	**9**	**-0.305**	**0.080**	***	**9**
	**EASTING**	**-0.231**	**0.081**	**	32	**-0.053**	**0.100**	***	**108**
	SLOPE_MEAN					-0.111	0.010	***	127
	WATER					2.776	0.519	***	14
Human	MGT_CORE	-2.434	0.184	***	287	-0.405	0.163		192
	MGT_REFUGE	-1.478	0.227	***		1.717	0.192	***	
	FEED	1.971	0.118	***	**366**				
	HUNT	1.048	0.247	***	**44**	-0.706	0.213	**	8
	**TOURI_W**	**-0.634**	**0.222**	**	**6**	**2.879**	**0.291**	***	**81**
	**ROAD**					**1.993**	**0.178**	***	**131**

Models explaining habitat use within the home range in summer (upper panel) and winter (lower panel) during daytime (left) and nighttime (right). For all variables positive estimates indicate preference, negative estimates indicate relative avoidance. For the predictors marked with bold letters the differences between daytime and nighttime habitat use were significant (see Table 5). For predictor names see Table 2. Significance levels are indicated with: * p ≤ 0.05, ** p ≤ 0.01, and *** p ≤ 0.001.

**Table 5:** Differences between diurnal and nocturnal habitat selection within the home range with regard to the relevant environmental predictors selected in the final models (Table 4).

	Summer (AUC = 0.895 +- 0.007)				Winter (AUC: 0.866 +- 0.011)		
	STD (Individual): 0.967				STD (Individual): 0.990		
Type	Variable	Estimate	SE	Sign.	Estimate	SE	Sign.
	INTERCEPT	6.661	0.322	***	6.431	0.448	***
Vegetation	CAN_CON	-1.194	0.448		-1.373	0.310	***
	CAN_DEC	-0.557	0.739		-4.467	2.293	*
	CAN_CONMIX	-1.074	0.447		-1.312	0.322	***
	CAN_DECMIX	-1.826	0.459	*	-3.483	0.615	***
	SUC_REGTHICK	-2.172	0.461	***			
	SUC_POLE	-1.234	0.454	***			
	SUC_TREE	-0.323	0.453	*			
	SUC_OLD	-0.341	0.464	*			
	BILBERRY	0.037	0.003	***			
	FOREST250	-2.207	0.250	***			
	UNDERCOV	-0.011	0.002	***			
Landscape	SLOPE	-0.065	0.005	***	-0.112	0.011	***
	WATER	4.110	0.314	***	10.742	0.651	***
	NORTHING	0.258	0.044	***			
	EASTING	-0.203	0.047	***			
Human	ROAD	0.740	0.068	***	2.015	0.230	***
	TOURI_S/W	1.733	0.222	***	8.290	0.478	***
	HUNT	-1.087	0.139	***	-2.689	0.310	***
	MGT_CORE	-0.269	0.208				
	MGT_REFUGE	0.187	0.220	.			

Positive estimates indicate a relatively more frequent use of this variable in the night, while negative estimates indicate a relative more frequent use during the day. Significance levels areindicated with: * p≤ 0.05 and *** p≤ 0.001.

## Discussion

### Effects of human presence and outdoor recreation

Our study shows how adjustments of behaviour can result in oppositional patterns of wildlife habitat use at day and nighttime, when areas frequented by recreationists are avoided during the day and preferred during the night. Although it is suggested that animals become habituated to human presence [73] and might reduce flight-distances in areas with frequent human-wildlife contact [74], the deer avoided the areas close to the trails during daytime. Whereas in North America it is a widely known phenomenon that deer habituate to humans and even occur in settlements where they are not hunted [51], the deer in our study seem to actively avoid human recreationists. This might indicate that red deer are unable to distinguish recreational users and hunters, and therefore temporally avoid areas with high human use. The diurnal pattern was blurred when pooling day and night locations (S2 Table), which highlights the importance of accounting for temporal differences when analyzing human-wildlife interactions.

Linking spatiotemporal patterns of wildlife habitat use to human presence is an important prerequisite for designing efficient wildlife management concepts, even if reducing disturbance is not the primary management goal as it might be the case in hunted species like the red deer. Previous studies showed that red deer respond to the presence of recreationists by fleeing [43], moving to denser vegetation areas [43], increasing vigilance [40] and adjusting their foraging behaviour [42]. Sibbald et al. [41] found an avoidance of hiking trails by red deer, which was stronger during the day with higher visitor numbers.

Since the infra-red counter data (S2 Fig), collected shortly after our study on red deer show a strong diurnal variation of use (i.e. many visitors during day, little or none during night) and no other factor in the area which is spatially linked to recreational trails shows a diurnal pattern, we assume that the avoidance of recreational trails by day is caused by a the presence of recreationists. Sibbald et al. [20], also showed that red deer flexibly adjust their habitat use to the diurnal variation in human presence. Even though we could not directly link deer behaviour to the intensity of recreation activities on the trails, as visitor counts obtained with photo sensors (S2 Fig) were collected one year after the telemetry data, we assume that the diurnal pattern of recreation activities was similar during the time of our study. However, detailed information on the number of visitors per specific trail and time of the day would be favorable for quantifying the number of visitors that triggers an avoidance reaction in red deer.

Apart from human recreational infrastructure, habitat selection was based on a variety of factors related to forage quality (e.g. bilberry, deciduous trees) and essential resources (e.g. water). The predominant use of these habitat features during night but not during the day indicates that resources attractive to the deer are temporally not accessible due to human disturbance. In our study area this applied to the area along the lake, which is highly frequented by recreationists, as well as to clearings with abundant ground vegetation and bilberry patches which are mainly located in open forest with high visibility. During the day, particularly in summer, the deer was more frequently found in dense forest stands providing cover, i.e. thickets and pole stands (Tables 4 and 5). This finding supports the suggestions by previous studies [44,47] that human disturbance may contribute to reinforce possible conflicts with forestry: if clearings and open forest near trails are not usable for foraging during the day due to disturbance, the deer may be forced to relocate to dense forest stands with cover where they may cause forest damage by tree browsing and bark stripping as no alternative food is available in these stands.

### Zoning scheme

To reduce human-wildlife conflicts, zoning schemes might play an important role for wildlife managers confronted with combining varying interests in human dominated landscapes within central Europe. In our study, both seasonal home range selection within the study area, as well as habitat use within the home range, were closely linked to the zoning scheme: red deer selected the refuge areas over the core and border zone of the management scheme and—as expected—stayed close to the feeding stations in winter. We cannot prove a causal effect of zoning on red deer habitat selection though, as no systematically collected data before the establishment of management zones were available and we cannot exclude that the delineation of zones might have been influenced by pre-existing expert knowledge. It is therefore possible that the deer had already preferred these areas prior to the establishment of the zonation scheme, due to other factors such as traditions or the distribution of forage.

### Management implications

The diurnal avoidance of human recreation infrastructure by red deer, both in summer and winter, associated with an increased nocturnal use of temporarily inaccessible resources has several implications for the management of natural areas. As the avoidance of trails during the daytime renders some areas and resources inaccessible to the deer, it is important that the animals are not additionally disturbed during the night. Nocturnal sport events (i.e. torch-lit walks, nocturnal orienteering) should thus be strictly regulated in areas with disturbance-sensitive wildlife. In addition, patches of open forest, clearings and meadows, providing alternative food sources should be created within sufficient distance and with visual protection from hiking trails. Wildlife refuges, from which recreation is banned provide undisturbed areas during both, day and night, and are likely to benefit also other disturbance-sensitive wildlife.

## Supporting information

S1 Data all relevant data for this study is included in the data file. (XLSX)

S1 Fig Relative probability of red deer presence in summer (upper panel) and winter (lower panel), during day (left) and night (right). Black and white represent high and low probability of presence respectively. Dashed lines indicate the presence of summer and winter recreation trails, respectively.
(DOCX)

S2 Fig Mean number of visitors per hour present on selected summer (black) and winter (grey) trails, for the different times of the day. The shaded area shows the time between sunrise and sunset for the studied time period. For three months (17.2.2010 14.4.2010) TRAFx Infrared trail counters were placed along three designated hiking trails and three cross country skiing trails within the study area. These count the number of times an individual passed the light sensor. Although we cannot exclude that red deer crossed the sensors, particularly during nighttime, we assume most of these crossings are humans since these were placed along designated recreation infrastructure. Most of the activity on the trails is during the time between sunrise and sunset.
(DOCX)

S1 Table Number of locations per individual used for analysis. Number of locations per individual per season and time of the day used for the analysis, and the period of tracking. The age was roughly estimated in three classes at the time of tagging (1 = 1–3 years, 2 = 3–5 years, 3 = >5 years old).
(DOCX)

S2 Table Models explaining habitat selection within the home range, when not discriminating between different times of the day. Left panel: summer, right panel: winter. Significance levels are indicated with: * p≤ 0.05, ** p≤ 0.01, and *** p≤ 0.001.
(DOCX)

S3 Table Results of the variable selection process to reach the final models presented in Tables 3 and 4. The first column shows all variables which were included in the model selection process. Each of the other columns represents one of the six final models: “HRinSA” = home range selection within study area, “inHR” = habitat selection within home range. Variables denominated with a “+” are included in the final model, otherwise the reason for exclusion is indicated: A variable name indicates exclusion due to pairwise correlation (Spearmans R >|0.5|) with this variable, “-”indicates exclusion during the model selection process based on AIC as described in the methods part, “VIF” indicates this variable is excluded from the model due to a too high variance inflation factor value. Proximity of feeding stations (FEED) was not included in the summer models as no feeding was performed in summer.
(DOCX)

S4 Table Final models (provided in Tables 3 and 4) in comparison to the next-best candidate models as obtained during the model selection process. All candidate models with a ΔAIC < 2 to the final model (in bold) as well as the first model with ΔAIC > 2 are shown. Only for the model describing home range selection in the study area during summer (Table 3A) the next four candidate models were not significantly different to the final model (i.e. ΔAIC < 2), so model averaging was applied (S5 Table).
(DOCX)

S5 Table Model averaging of the model describing home range selection in study area during summer (Table 3A). The table shows the variables included in the five component models (with variable codes described below), as well as the relative importance of the variables.
(DOCX)

S6 Table Variation inflation factors (VIF) for the variables included in the models presented in Tables 3 and 4. For categorical variables the corrected VIF values (VIF^(1/2Df)) are provided.
(DOCX)

S7 Table Odd’s ratios with 95% confidence interval for the coefficients of the models provided in Tables 3 and 4. Odd’s ratios were obtained using the Wald chi-square method, the lower (2.5%) and upper (97.5%) boundary of the confidence interval are provided.
(DOCX)

## References

[1] Stankowich T. Ungulate flight responses to human disturbance: a review and meta-analysis. Biol Conserv. 2008;141: 2159–2173.

[2] Flather CH, Cordell HK. Outdoor Recreation: Historical and Anticipated Trends In: Knight RL, Gutzwiller KJ, editors. Wildlife and recreationists: coexistence through management. Washington DC, USA: Island Press; 1995.

[3] Jiang G, Ma J, Zhang M, Stott P. Effects of human activities on the spatial distribution of eastern roe deer *Capreolus pygargus bedfordi* in the Lesser Khingan Mountains, northeastern China. Acta Theriol. 2009;54(1): 61–76.

[4] Reimoser S. Influence of anthropogenic disturbances on activity, behaviour and heart rate of roe deer (*Capreolus capreolus*) and red deer (*Cervus elaphus*), in context of their daily and yearly patterns In: Cahler AA, Marsten JP, editors. Deer: Habitat, Behavior and Conservation. New York, USA: Nova Science Publishers Inc; 2012.

[5] Yasué M. Environmental factors and spatial scale influence shorebirds' responses to human disturbance. Biol Cons. 2005;128: 47–54.

[6] Leblond M, Dussault C, Ouellet J-P. Impacts of human disturbance on large prey species: Do behavioral reactions translate to fitness consequences? PLoS ONE. 2013; 8(9): e73695 doi: 10.1371/journal.pone.0073695 24040029 10.1371/journal.pone.0073695 PMC3770704

[7] Frid A, Dill L. Human-caused disturbance stimuli as a form of predation risk. Cons Ecol. 2002;6(1): 11p. [online] URL: http://www.consecol.org/vol6/iss1/art11/

[8] Beale CM, Monaghan P. Human disturbance: people as predation-free predators? J. Appl Ecol. 2004;41: 335–343.

[9] Knight RL, Temple SA. Wildlife and recreationists: coexistence through management In: Knight RL, Gutzwiller KJ, editors. Wildlife and recreationists: coexistence through management. Washington DC, USA: Island Press; 1995.

[10] Knight RL, Cole DN. Wildlife responses to recreationists In: Knight RL, Gutzwiller KJ, editors. Wildlife and recreationists: coexistence through management. Washington DC, USA: Island Press; 1995.

[11] Thiel D, Jenni-Eiermann S, Braunisch V, Palme R, Jenni L. Ski tourism affects habitat use and evokes a physiological stress response in capercaillie *Tetrao urogallus*: a new methodological approach. J Appl Ecol. 2008; 45: 845–853.

[12] Formenti N, Viganó R, Bionda R, Ferrari N, Trogu T, Lanfranchi P, Palme R. Increased hormonal stress reactions induced in an Alpine Black Grouse (*Tetrao tetrix*) population by winter sports. J Ornithol. 2015;156(1): 317–321.25363236

[13] Arlettaz R, Patthey P, Baltic M, Leu T, Schaub M, Palme R, Jenni-Eiermann S. Spreading free-riding snow sports represent a novel serious threat for wildlife. Proc R Soc B. 2007;274: 1219–1224. doi: 10.1098/rspb.2006.0434 17341459 10.1098/rspb.2006.0434 PMC2189568

[14] Sheriff MJ, Krebs CJ, Boonstra R. The sensitive hare: sublethal effects of predator stress on reproduction in snowshoe hares. J Anim Ecol. 2009;78: 1249–1258. doi: 10.1111/j.1365-2656.2009.01552.x 19426257 10.1111/j.1365-2656.2009.01552.x

[15] Fernández-Juricic E, Telleria JL. Effects of human disturbance on spatial and temporal feeding patterns of blackbird *Turdus merula* in urban parks in Madrid, Spain. Bird Study. 2000;47: 13–21.

[16] Miller SG, Knight RL, Miller CK. Wildlife responses to pedestrians and dogs. Wildl Soc Bull. 2001;29(1): 124–132.

[17] Sönnichsen L, Bokje M, Marchal J, Hofer H, Jędrzejewska B, Kramer-Schadt S, et al Behavioural Responses of European Roe Deer to Temporal Variation in Predation Risk. Ethol. 2013;199(3): 233–243.

[18] Taylor AR, Knight RL. Wildlife responses to recreation and associated visitor perceptions. Ecol Appl. 2003;13(4): 951–963.

[19] Immitzer M, Nopp-Mayr U, Zohmann M. Effects of habitat quality and hiking trails on the occurrence of Black Grouse (*Tetrao tetrix L*.) at the northern fringe of alpine distribution in Austria. J Ornithol. 2014;155(1): 173–181.

[20] Sibbald AM, Hooper RJ, McLeod JE, Gordon IJ. Responses of red deer (*Cervus elaphus*) to regular disturbance by hill walkers. Eur J Wildl Res. 2011;57:817–825.

[21] Phillips GE, Alldredge AW. Reproductive success of elk following disturbance by humans during calving season. J Wildl Manage. 2000; 64(2): 521–530.

[22] Arnold W, Ruf T, Reimoser S, Tataruch F, Onderscheka K, Schober F. Nocturnal hypometabolism as an overwintering strategy of red deer (*Cervus elaphus*). Am J Physiol Regul Integr Comp Physiol. 2004;268: 174–181. 10.1152/ajpregu.00593.2002 12969877

[23] Ciuti S, Northrup JM, Muhly TB, Simi S, Musiani M, Pitt JA, Boyce MS. Effects of Humans on Behaviour of Wildlife Exceed Those of Natural Predators in a Landscape of Fear. PLoS ONE. 2012;7(11): e50611 doi: 10.1371/journal.pone.0050611 23226330 10.1371/journal.pone.0050611 PMC3509092

[24] Lima SL, Blackwell BF, Travis L. DeVault TL, Fernández-Juricic E. Animal reactions to oncoming vehicles: a conceptual review. Biol Rev. 2015;90: 60–76. doi: 10.1111/brv.12093 24661508 10.1111/brv.12093

[25] Reimoser F. Weniger Wildschäden durch Ruhezonen? Öster Forstz. 1988; 24–25. German.

[26] Jeppesen JL. Impact of human disturbance on home range, movements and activity of red deer (*Cervus elaphus*) in a Danish environment. Danish Rev Game Biol. 1987; 13(2): 1–35.

[27] Dudley N, editor.Guidelines for Applying Protected Area Management Categories. IUCN Gland, Switzerland 2008.

[28] Milner JM, Bonenfant C, Mysterud A, Gaillard J, Csanyi S, Stenseth NC. Temporal and spatial development of red deer harvesting in Europe: biological and cultural factors. J Appl Ecol. 2006;43(4): 721–734.

[29] Koubek P, Zima J. Cervus elaphus In: Mitchell-Jones AJ, Amori G, Bogdanowicz W, Kryßtufek B, Reijnders PJH, Spitzen-berger F, et al, editors. The atlas of European mammals. London, UK Academic Press; 1999.

[30] Bützler W. Rotwild: Biologie, Verhalten, Umwelt, Hege. Munich, Germany: BLV; 2001. German.

[31] Putman R, Apollonio M, Andersen R. Ungulate Management in Europe: Problems and practices. Cambridge, UK Cambridge University Press; 2011.

[32] von Oheimb G, Schmidt M, Kriebitzsch W-U, Ellenberg H. Dispersal of vascular plants by game in northern Germany. Part II: Red deer (*Cervus elaphus*). Eur J For Res. 2005;124(1): 55–65.

[33] Iravani M, Schuetz M, Edwards PJ, Risch AC, Scheidegger C, Wagner HH. Seed dispersal in red deer dung and its importance for vegetation dynamics in subalpine grasslands. Basic Appl Ecol. 2011;12: 505–515.

[34] Ruiz-Fons F, Gilbert L. The role of deer as vehicles to move ticks, *Ixodes ricinus* between contrasting habitats. Int J Parasit. 2010; 40: 1013–1020. 10.1016/j.ijpara.2010.02.006 20211625

[35] Jȩdrzejewski W, Jȩdrzejewska B, Okarma H, Schmidt K, Zub K, Musiani M. Prey selection and predation by wolves in Białowieża Primeval Forest, Poland. J Mammal. 2000;81(1): 197–212.

[36] Gill RMA, Beardall V. The impact of deer on woodlands: the effects of browsing and seed dispersal on vegetation structure and composition. Forestry. 2001;74(3): 209–218.

[37] Côté SD, Thomas P, Rooney TP, Tremblay J-P, Dussault C, Waller DM. Ecological impacts of deer overabundance. Ann Rev Ecol Evol Syst. 2004;35(1): 113–147.

[38] Melis C, Buset A, Aarrestad PA, Hanssen O, Meisingset EL, Andersen R, et al) Impact of red deer *Cervus elaphus* grazing on bilberry *Vaccinium myrtillus* and composition of ground beetle (*Coleoptera*, *Carabidae*) assemblage. Biodiv Cons. 2005;15(6): 2249–2259.

[39] Hegland SJ, Lilleeng MS, Moe SR. Old-growth forest floor richness increases with red deer herbivory intensity. For Ecol Manage. 2013;310: 267–274.

[40] Gill RMA. A review of damage by mammals in north temperate forests: 3. impact on trees and forests. Forestry. 1992;65: 363–388.

[41] Kiffner C, Rössiger E, Trisl O, Schulz R, Rühe F. Probability of recent bark stripping damage by red deer (*Cervus elaphus*) on Norway spruce (*Picea abies*) in a low mountain range in Germany–a preliminary analysis. Silva Fenn. 2008;42: 125–134.

[42] Groot Bruinderink GWTA, Hazebroek E. Ungulate Traffic Collisions in Europe. Cons Biol. 1996;10(4): 1059–1067.

[43] Gunson KE, Mountrakis G, Quackenbush LJ. Spatial wildlife-vehicle collision models: a review of current work and its application to transportation mitigation projects. J Environ Manage. 2011;92: 1074–1082. doi: 10.1016/j.jenvman.2010.11.027 21190788 10.1016/j.jenvman.2010.11.027

[44] Seiler A. Trends and spatial patterns in ungulate-vehicle collisions in Sweden. Wildl Biol. 2004;10: 301–313.

[45] Morellet N, Gaillard JM, Hewison AJM, Ballon P, Boscardin Y, Duncan P, et al Indicators of ecological change: new tools for managing populations of large herbivores. J Appl Ecol. 2007;44: 634–643.

[46] Mysterud A, Tryjanowski P, Panek M. Selectivity of harvesting differs between local and foreign roe deer hunters: trophy stalkers have the first shot at the right place. Biol Lett. 2006;2: 632–635. doi: 10.1098/rsbl.2006.0533 17148307 10.1098/rsbl.2006.0533 PMC1833986

[47] Jayakody S, Sibbald AM, Gordon IJ, Lambin X. Red deer *Cervus elaphus* vigilance behaviour differs with habitat and type of human disturbance. Wildl Biol. 2008;14(1): 81–91.

[48] Jayakody S, Sibbald AM, Mayes RW, Hooper RJ, Gordon IJ, Lambin X. Effects of human disturbance on the diet composition of wild red deer (*Cervus elaphus*). Eur J Wildl Res. 2011;57: 939–948.

[49] Burghardt F, Hagen R, Heurich M, Rummel A, Suchant R. Reaktionen unbejagter Rothirsche eines Nationalparks und Reaktion von Rothirschen einer intensiv bejagten Population auf Freizeitaktivitäten abseits ausgewiesener Wege. Freiburg, Germany FVA 2012. German.

[50] Lone K, Loe LE, Meisingset EL, Stamnes I, Mysterud A. An adaptive behavioural response to hunting: surviving male red deer shift habitat at the onset of the hunting season. Anim Behav. 2015;102: 127–138.

[51] Thompson M, Henderson R. Elk habituation as a credibility challenge for wildlife professionals. Wildl Soc Bull. 1998;26(3): 477–483.

[52] Lubow BC, Singer FJ, Johnson TL, Bowden DC. Dynamics of interacting elk populations within and adjacent to Rocky Mountain National Park. J Wildl Manage. 2002;66(3): 757–775.

[53] Staines BW, Welch D. Impact of red and roe deer on Scottish woodlands In: Putman RJ, editor. Mammals as pests: Chapman and Hall, Edinburgh, UK 1989; 128–130.

[54] Kloppers EL, St Clair CC, Hurd TE. Predator-resembling aversive conditioning for managing habituated wildlife. Ecol Soc. 2005;10(1): 31 [online] URL: http://www.ecologyandsociety.org/vol10/iss1/art31/

[55] Putman RJ, Staines BW. Supplementary winter feeding of wild red deer *Cervus elaphus* in Europe and North America: justifications, feeding practice and effectiveness. Mamm Rev. 2004;34(4): 285–306.

[56] Suchant R, Burghardt F, Gerecke KL. Rotwild im Südschwarzwald Konzeption eines integrativen Rotwild-Managements. Freiburg, Germany Projektgruppe Rotwild 2008. German.

[57] Miller AD, Vaske JK, Squires JR, Olson LE, Roberts EK. Does zoning winter recreationists reduce recreation conflict? Envir Manage. 2017;59: 50–67. 10.1007/s00267-016-0777-0 27734085

[58] Krishna YC, Kumar A, Isvaran K. Wild ungulate decision-making and the role of tiny refuges in human-dominated landscapes. PLoS ONE. 2016;11(2): e0151748. 26985668 10.1371/journal.pone.0151748 PMC4795686

[59] Fernandez-Juricic E, Vaca R, Schroeder N. Spatial and temporal responses of forest birds to human approaches in a protected area and implications for two management strategies. Biol Cons. 2004;117(4): 407–416.

[60] Suchant R, Baritz R, Braunisch V. Wildlife habitat analysis—a multidimensional habitat management model. J Nat Cons. 2003;10(4): 253–268.

[61] Statistisches Landesamt. 2016. Tourismusentwicklung absolut 2004–2015 nach Gebieten und Gesamtschwarzwald. http://www.schwarzwald-tourismus.info/partnernet/Interne-Infos/Marktforschung-Statistiken/Statistiken-vom-Statistischen-Landesamt/Tourismusentwicklung-2004-05-2015. German.

[62] Coppes J, Braunisch V. Managing visitors in nature areas: where do they leave the trails? A spatial model. Wildl Biol. 2013;19(1): 1–11.

[63] Stache A, Löttker P, Heurich M. Red deer telemetry: Dependency of the position acquisition rate and accuracy of GPS collars on the structure of a temperate forest dominated by European beech and Norway spruce. Silv Gabr. 2012;18(1): 35–48.

[64] Johnson DH. The comparison of usage and availability measurements for evaluating resource preference. Ecol. 1980;61(1): 65–71.

[65] Bates D, Maechler M, Bolker B, Walker S. lme4: Linear mixed-effects models using Eigen and S4. 2014. R package version 1.1–8, http://CRAN.R-project.org/package=lme4.

[66] Bartoń K. MuMIn: multi-model inference. 2014. R package version 1.9.13 http://CRAN.R-project.org/package=MuMIn.

[67] Burnham KP, Anderson DR. Model Selection and Inference. New York, USA: Springer-Verlag 1998.

[68] Zuur AF, Ieno EN, Walker N, Saveliev AA, Smith GM. Mixed effects models and extensions in ecology with R. New York, USA Springer 2009.

[69] Marquaridt DW. Generalized inverses, ridge regression, biased linear estimation, and nonlinear estimation. Technometrics. 1970;12(3): 591–612.

[70] Fox J. Applied regression analysis, linear models, and related methods: Thousand Oaks, USA Sage Publications 1997.

[71] Mazerolle MJ. AICcmodavg: Model selection and multimodel inference based on (Q)AIC(c). 2014. R package version 2.0–3. http://CRAN.R-project.org/package=AICcmodavg.

[72] Hosmer DWJ, Lemeshow S. Applied Logistic Regression, 2nd edition New York, USA John Wiley & Sons 2000.

[73] Picton HD. Energetic cost of wildlife displacement by winter recreationists. In: Olliff T, Legg K, Kaeding B, editors. Effects of winter recreation on wildlife of the Greater Yellowstone Area: a literature review and assessment. Yellowstone National Park, Wyoming: Report to the Greater Yellowstone Coordinating Committee. 2000.

[74] Stankowich T, Blumstein DT. Fear in animals: a meta-analysis and review of risk assessment. Proc R Soc B. 2005;272: 2627–2634. doi: 10.1098/rspb.2005.3251 16321785 10.1098/rspb.2005.3251 PMC1559976

