# Supplementary material for: Human recreation affects spatio-temporal habitat use patterns in red deer (Cervus elaphus)
Source: PLoS One. 2017 May 3;12(5):e0175134. doi: 10.1371/journal.pone.0175134 (PMC5414982; doi:10.1371/journal.pone.0175134)
Supplement: S1 Fig — Black and white represent high and low probability of presence respectively. Dashed lines indicate the presence of summer and winter recreation trails, respectively. (DOCX) [file pone.0175134.s002.docx]

Supporting Information PONE-D-16-42033R2

**Coppes et al. 2017: Human recreation affects spatio-temporal habitat use patterns in red deer (Cervus elaphus)**

**S1 Fig.** **Relative probability of red deer presence** **in summer (upper panel) and winter (lower panel), during day (left) and night (right).** Black and white represent high and low probability of presence respectively. Dashed lines indicate the presence of summer and winter recreation trails, respectively.
