# Supplementary material for: Human recreation affects spatio-temporal habitat use patterns in red deer (Cervus elaphus)
Source: PLoS One. 2017 May 3;12(5):e0175134. doi: 10.1371/journal.pone.0175134 (PMC5414982; doi:10.1371/journal.pone.0175134)

Supporting Information PONE-D-16-42033R2

**Coppes et al. 2017: Human recreation affects spatio-temporal habitat use patterns in red deer (Cervus elaphus)**

**S2 Fig.** **Mean number of visitors per hour present on selected summer (black) and winter (grey) trails, for the different times of the day.** The shaded area shows the time between sunrise and sunset for the studied time period. For three months (17.2.2010 14.4.2010) TRAFx Infrared trail counters were placed along three designated hiking trails and three cross country skiing trails within the study area. These count the number of times an individual passed the light sensor. Although we cannot exclude that red deer crossed the sensors, particularly during nighttime, we assume most of these crossings are humans since these were placed along designated recreation infrastructure. Most of the activity on the trails is during the time between sunrise and sunset.


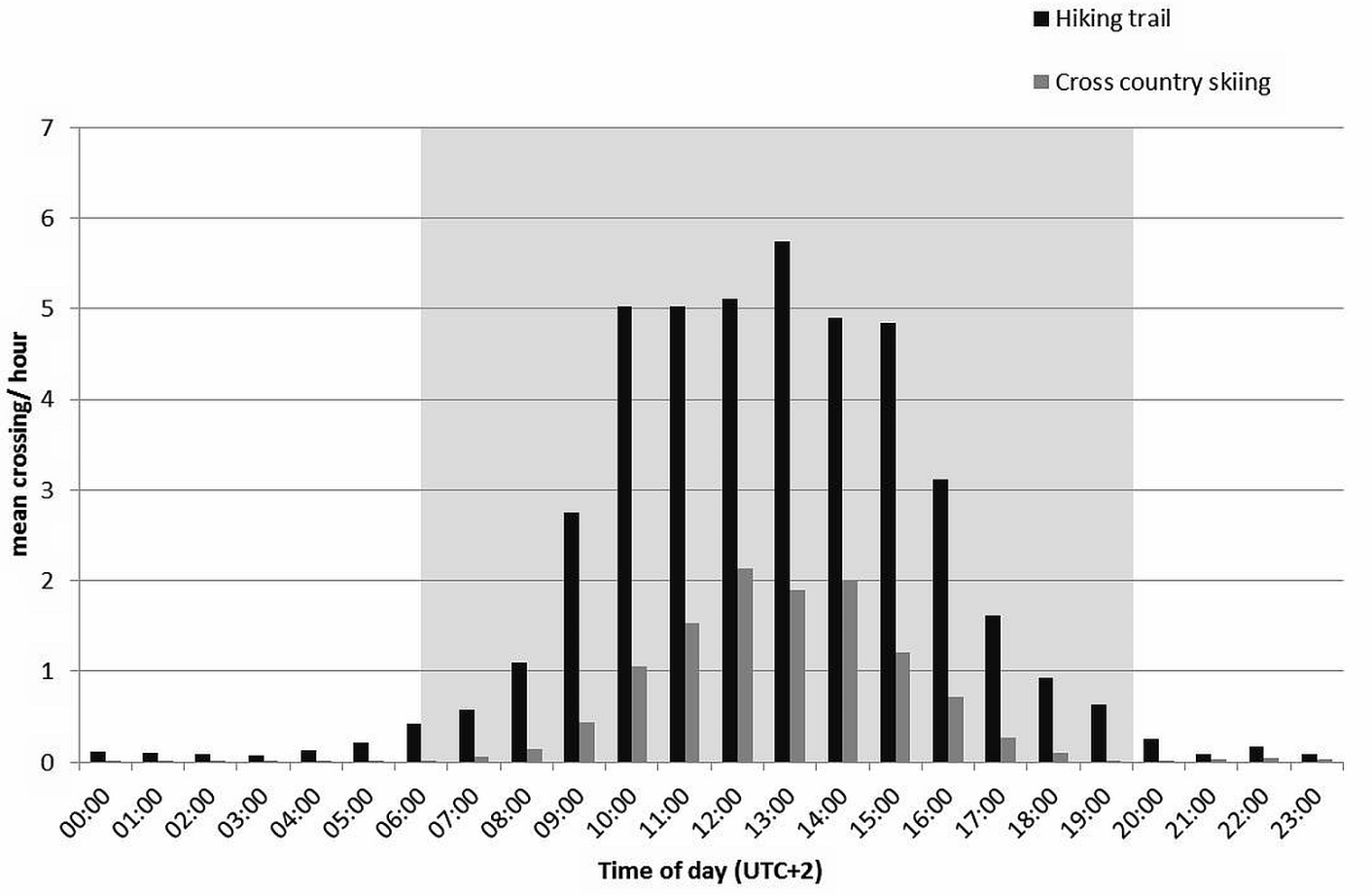

Supplement: S2 Fig — The shaded area shows the time between sunrise and sunset for the studied time period. For three months (17.2.2010 14.4.2010) TRAFx Infrared trail counters were placed along three designated hiking trails and three cross country skiing trails within the study area. These count the number of times an individual passed the light sensor. Although we cannot exclude that red deer crossed the sensors, particularly during nighttime, we assume most of these crossings are humans since these were placed along designated recreation infrastructure. Most of the activity on the trails is during the time between sunrise and sunset. (DOCX) [file pone.0175134.s003.docx]
