# Supplementary material for: Human recreation affects spatio-temporal habitat use patterns in red deer (Cervus elaphus)
Source: PLoS One. 2017 May 3;12(5):e0175134. doi: 10.1371/journal.pone.0175134 (PMC5414982; doi:10.1371/journal.pone.0175134)
Supplement: S1 Table — Number of locations per individual per season and time of the day used for the analysis, and the period of tracking. The age was roughly estimated in three classes at the time of tagging (1 = 1–3 years, 2 = 3–5 years, 3 = >5 years old). (DOCX) [file pone.0175134.s004.docx]

Supporting Information PONE-D-16-42033R2

**Coppes et al. 2017: Human recreation affects spatio-temporal habitat use patterns in red deer (Cervus elaphus)**

**S1 Table. Number of locations per individual used for analysis.** Number of locations per individual per season and time of the day used for the analysis and the period of tracking. The age was roughly estimated in three classes at the time of tagging (1 = 1-3 years, 2 = 3-5 years, 3 = >5 years old).

|  |  | Summer | |  |  | Winter | |  |  |
| --- | --- | --- | --- | --- | --- | --- | --- | --- | --- |
| Individual |  |  |  |  |  |  |  |  | Months |
| ID | Age | Total | Day | Night |  | Total | Day | Night | tracked |
| Females |  |  |  |  |  |  |  |  |  |
| 101 | 1 | 818 | 411 | 407 |  | 2169 | 1004 | 1165 | 29 |
| 103 | 2 | 525 | 261 | 264 |  | 671 | 295 | 376 | 22 |
| 104 | 2 | 341 | 170 | 171 |  | 999 | 468 | 531 | 16 |
| 105 | 3 | 348 | 185 | 163 |  | 945 | 430 | 515 | 18 |
| 106 | 1 | 485 | 241 | 244 |  | 1452 | 633 | 819 | 23 |
| 107 | 1 | 527 | 263 | 264 |  | 1572 | 700 | 872 | 24 |
| 108 | 1 | 521 | 261 | 260 |  | 1637 | 709 | 928 | 24 |
| 109 | 1 | 518 | 261 | 257 |  | 1389 | 605 | 784 | 22 |
| 110 | 1 | 149 | 75 | 74 |  | 95 | 35 | 60 | 5 |
| 111 | 1 | 268 | 135 | 133 |  | 137 | 126 | 11 | 9 |
| Males |  |  |  |  |  |  |  |  |  |
| 201 | 3 | 302 | 150 | 152 |  | 223 | 34 | 189 | 12 |
| 203 | 1 | 564 | 282 | 282 |  | 1847 | 782 | 1065 | 25 |
| 204 | 1 | 825 | 413 | 412 |  | 2311 | 1052 | 1259 | 34 |
| 205 | 1 | 683 | 348 | 335 |  | 628 | 241 | 387 | 28 |
| 206 | 1 | 510 | 253 | 257 |  | 834 | 358 | 476 | 20 |
