# Supplementary material for: Human recreation affects spatio-temporal habitat use patterns in red deer (Cervus elaphus)
Source: PLoS One. 2017 May 3;12(5):e0175134. doi: 10.1371/journal.pone.0175134 (PMC5414982; doi:10.1371/journal.pone.0175134)
Supplement: S2 Table — Left panel: summer, right panel: winter. Significance levels are indicated with: * p≤ 0.05, ** p≤ 0.01, and *** p≤ 0.001. (DOCX) [file pone.0175134.s005.docx]

Supporting Information PONE-D-16-42033R2

**Coppes et al. 2017: Human recreation affects spatio-temporal habitat use patterns in red deer (Cervus elaphus)**

**S2 Table**. **Models explaining habitat selection within the home range, when not discriminating**

**between different times of the day**. Left panel: summer, right panel: winter. Significance levels are indicated with: * p≤ 0.05, ** p≤ 0.01, and *** p≤ 0.001.

|  | Summer (AUC = 0.624 +- 0.007) | | | | | Winter (AUC: 0.803 +- 0.008) | | |
| --- | --- | --- | --- | --- | --- | --- | --- | --- |
|  | STD (Individual): 0.041 | | | | | STD (Individual): 0.462 | | |
| Type | Variable | Estimate | SE | Sign. |  | Estimate | SE | Sign. |
|  | INTERCEPT | 0.296 | 0.102 | ** |  | -1.164 | 0.466 | ** |
| Vegetation | CAN_CON | -0.566 | 0.253 | * |  | 0.571 | 0.471 |  |
|  | CAN_DEC | -0.575 | 0.383 |  |  | 0.080 | 1.010 |  |
|  | CAN_CONMIX | -0.463 | 0.253 |  |  | 0.267 | 0.473 |  |
|  | CAN_DECMIX | -0.466 | 0.259 |  |  | -0.257 | 0.495 |  |
|  | SUC_REG_THICK | 1.635 | 0.261 | *** |  | 0.907 | 0.479 | . |
|  | SUC_POLE | 1.011 | 0.254 | *** |  | -0.020 | 0.472 |  |
|  | SUC_TREE | 0.726 | 0.252 | ** |  | 0.672 | 0.469 |  |
|  | SUC_OLD | 0.594 | 0.255 | * |  | 1.020 | 0.475 | * |
|  | UNDER_CON | 0.291 | 0.063 | *** |  |  |  |  |
|  | UNDER_DEC | 1.208 | 0.159 | *** |  |  |  |  |
|  | UNDER_CONMIX | 0.489 | 0.049 | *** |  |  |  |  |
|  | UNDER_DECMIX | 0.063 | 0.058 |  |  |  |  |  |
|  | CANOPY_COVER | -0.024 | 0.001 | *** |  |  |  |  |
|  | PROTECT_S | -0.005 | 0.001 | *** |  |  |  |  |
| Landscape | EASTING | -0.106 | 0.025 | *** |  | -0.492 | 0.048 | *** |
|  | SLOPE | 0.012 | 0.003 | *** |  | -0.030 | 0.005 | *** |
|  | ALTITUDE |  |  |  |  | 0.003 | <0.001 | *** |
| Human | MGT_CORE | 1.564 | 0.072 | *** |  | -0.669 | 0.092 | *** |
|  | MGT_REFUGE | 1.908 | 0.083 | *** |  | 0.447 | 0.118 | *** |
|  | ROAD | 0.602 | 0.038 | *** |  | 1.405 | 0.094 | *** |
|  | TOURI_S/W | -0.498 | 0.116 | *** |  | 0.722 | 0.139 | *** |
|  | HUNT | 0.651 | 0.065 | *** |  |  |  |  |
|  | SETTLE | 0.061 | 0.031 | * |  |  |  |  |
|  | FEED |  |  |  |  | 1.276 | 0.056 | *** |
