# Supplementary material for: Human recreation affects spatio-temporal habitat use patterns in red deer (Cervus elaphus)
Source: PLoS One. 2017 May 3;12(5):e0175134. doi: 10.1371/journal.pone.0175134 (PMC5414982; doi:10.1371/journal.pone.0175134)
Supplement: S3 Table — The first column shows all variables which were included in the model selection process. Each of the other columns represents one of the six final models: “HRinSA” = home range selection within study area, “inHR” = habitat selection within home range. Variables denominated with a “+” are included in the final model, otherwise the reason for exclusion is indicated: A variable name indicates exclusion due to pairwise correlation (Spearmans R >|0.5|) with this variable, “-”indicates exclusion during the model selection process based on AIC as described in the methods part, “VIF” indicates this variable is excluded from the model due to a too high variance inflation factor value. Proximity of feeding stations (FEED) was not included in the summer models as no feeding was performed in summer. (DOCX) [file pone.0175134.s006.docx]

Supporting Information PONE-D-16-42033R2

**Coppes et al. 2017: Human recreation affects spatio-temporal habitat use patterns in red deer (Cervus elaphus)**

**S3 Table: Results of the variable selection process** **to reach the final models presented in Table 3 and 4.** The first column shows all variables which were included in the model selection process. Each of the other columns represents one of the six final models: “HRinSA” = home range selection within study area, “inHR” = habitat selection within home range. Variables denominated with a “+” are included in the final model, otherwise the reason for exclusion is indicated: A variable name indicates exclusion due to pairwise correlation (Spearmans R >|0.5|) with this variable, “-“ indicates exclusion during the model selection process based on AIC as described in the methods part, “VIF” indicates this variable is excluded from the model due to a too high variance inflation factor value. Proximity of feeding stations (FEED) was not included in the summer models as no feeding was performed in summer.

| Variables tested | HRinSA summer | HRinSA winter | inHR summer day | inHR summer night | inHR winter day | In HR winter night |
| --- | --- | --- | --- | --- | --- | --- |
| DHM | - | SETTLE | SETTLE | GREENL | - | - |
| SLOPE | + | - | + | + | - | + |
| NORTHING | - | + | + | - | + | + |
| EASTING | - | + | + | + | + | + |
| WATER | + | + | + | + | - | + |
| GREENL | FOREST250 | - | FOREST_250 | ROAD | - | - |
| FOREST_250 | + | - | + | ROAD | - | - |
| CANOPY_TYPE | + | - | - | + | + | - |
| CANOPY_COV | HERB_GRAS | + | + | + | + | VIF |
| SUCCESSION | + | + | + | + | - | + |
| UNDER_COV | + | - | - | - | PROTECTION | - |
| UNDER_TYPE | - | - | + | - | - | - |
| BILBERRY | + | - | + | + | - | - |
| HERB_GRAS | + | - | CANOPY_COV | CANOPY_COV | - | - |
| PROTECTION_S/W | + | + | + | CANOPY_COV | - | + |
| TOURI_S/W | + | - | + | + | + | + |
| TOURI_DENS_S/W | TOURI_S/W | TOURI_S/W | TOURI_S/W | TOURI_S/W | TOURI_S/W | TOURI_S/W |
| ROAD | + | + | - | + | - | + |
| SETTLE | + | + | - | + | - | - |
| FEED | Not included | + | Not included | Not included | + | + |
| HUNT | + | + | + | + | + | + |
| CONCEPT | + | + | + | + | + | + |
