# Supplementary material for: Human recreation affects spatio-temporal habitat use patterns in red deer (Cervus elaphus)
Source: PLoS One. 2017 May 3;12(5):e0175134. doi: 10.1371/journal.pone.0175134 (PMC5414982; doi:10.1371/journal.pone.0175134)
Supplement: S4 Table — All candidate models with a ΔAIC < 2 to the final model (in bold) as well as the first model with ΔAIC > 2 are shown. Only for the model describing home range selection in the study area during summer (Table 3A) the next four candidate models were not significantly different to the final model (i.e. ΔAIC < 2), so model averaging was applied (S5 Table). (DOCX) [file pone.0175134.s007.docx]

Supporting Information PONE-D-16-42033R2

**Coppes et al. 2017: Human recreation affects spatio-temporal habitat use patterns in red deer (Cervus elaphus)**

**S4 Table: Final models (provided in Tables 3 and 4) in comparison to the next-best candidate models as obtained during the model selection process** All candidate models with a ΔAIC < 2 to the final model (in bold) as well as the first model with ΔAIC > 2 are shown. Only for the model describing home range selection in the study area during summer (Table 3a) the next four candidate models were not significantly different to the final model (i.e. ΔAIC < 2), so model averaging was applied (Table S5).

|  | Selection of home range in study area: summer | | | | |  | |  |  | | |  |  | |  | |  | |  | |  |  |  |  |  |
| --- | --- | --- | --- | --- | --- | --- | --- | --- | --- | --- | --- | --- | --- | --- | --- | --- | --- | --- | --- | --- | --- | --- | --- | --- | --- |
| NR | INTERCEPT | BILBERRY | CANOPY TYPE | FOREST250 | MGT | HERB_GRAS | HUNT | | PROTECT_S | SETTLE | SLOPE | | SUCCESSION | TOURI_S | | WATER | |  | df | logLik | AIC | | | delta | |
| **2752** | **-1.9042** | **-0.0084** | **+** | **0.5254** | **+** | **0.0175** | **-0.0018** | | **NA** | **0.0003** | **NA** | | **+** | **NA** | | **-0.0011** | |  | **18** | **-12087.5** | **24210.9** | | | **0.00** | |
| **3008** | **-1.8626** | **-0.0084** | **+** | **0.5256** | **+** | **0.0174** | **-0.0018** | | **NA** | **0.0003** | **-0.0033** | | **+** | **NA** | | **-0.0012** | |  | **19** | **-12086.8** | **24211.7** | | | **0.71** | |
| **4032** | **-1.8453** | **-0.0085** | **+** | **0.5595** | **+** | **0.0176** | **-0.0018** | | **NA** | **0.0003** | **-0.0043** | | **+** | **-0.0002** | | **-0.0011** | |  | **20** | **-12086.0** | **24212.0** | | | **1.05** | |
| **3776** | **-1.9002** | **-0.0085** | **+** | **0.5501** | **+** | **0.0176** | **-0.0018** | | **NA** | **0.0003** | **NA** | | **+** | **-0.0001** | | **-0.0011** | |  | **19** | **-12087.0** | **24212.0** | | | **1.06** | |
| **2816** | **-1.9048** | **-0.0084** | **+** | **0.5257** | **+** | **0.0175** | **-0.0018** | | **0.0000** | **0.0003** | **NA** | | **+** | **NA** | | **-0.0011** | |  | **19** | **-12087.5** | **24212.9** | | | **2.00** | |
| 3072 | -1.8618 | -0.0084 | + | 0.5253 | + | 0.0174 | -0.0018 | | 0.0000 | 0.0003 | -0.0033 | | + | NA | | -0.0012 | |  | 20 | -12086.8 | 24213.7 | | | 2.70 | |
|  |  |  |  |  |  |  |  | |  |  |  | |  |  | |  | |  |  |  |  | | |  | |
|  | Selection of home range in study area: winter | | | | |  |  | |  |  |  | |  |  | |  | |  |  |  |  | | |  | |
| NR | INTERCEPT | EASTING | FEED | HUNT | MGT | NORTHING | PROTECT_W | | ROAD | SETTLE | SUCCESSION | | TREECOVER | WATER | |  | |  | df | logLik | AIC | | | delta | |
| **2048** | **2.9768** | **-1.0539** | **-0.0021** | **-0.0019** | **+** | **-0.2697** | **-0.0100** | | **-0.0007** | **-0.0004** | **+** | | **-0.0106** | **0.0009** | |  | |  | **17** | **-4140.4** | **8314.9** | | | **0.00** | |
| 1024 | 3.0491 | -1.0648 | -0.0020 | -0.0019 | + | -0.2740 | -0.0094 | | -0.0007 | -0.0004 | + | | -0.0100 | NA | |  | |  | 16 | -4144.9 | 8321.8 | | | 6.91 | |
|  |  |  |  |  |  |  |  | |  |  |  | |  |  | |  | |  |  |  |  | | |  | |
|  | Selection in home range during summer: day | | | | |  |  | |  |  |  | |  |  | |  | |  |  |  |  | | |  | |
| NR | INTERCEPT | BILBERRY | EASTING | FOREST250 | HUNT | MGT | NORTHING | | PROTECT_S | WATER | SUCCESSION | | TOURI_S | CANOPY_COV | | UNDER_TYPE | | SLOPE | df | logLik | AIC | | | delta | |
| **8192** | **-4.6754** | **-0.0246** | **0.0833** | **1.5835** | **-0.0012** | **+** | **-0.2787** | | **-0.0040** | **0.0025** | **+** | | **0.0016** | **-0.0128** | | **+** | | **0.0637** | **22** | **-5997.9** | **12039.8** | | | **0.00** | |
| 8190 | -4.6217 | -0.0248 | NA | 1.5719 | -0.0012 | + | -0.2845 | | -0.0038 | 0.0024 | + | | 0.0016 | -0.0130 | | + | | 0.0639 | 21 | -5999.9 | 12041.8 | | | 2.02 | |
|  |  |  |  |  |  |  |  | |  |  |  | |  |  | |  | |  |  |  |  | | |  | |
|  | Selection in home range during summer: night | | | | |  |  | |  |  |  | |  |  | |  | |  |  |  |  | | |  | |
| NR | INTERCEPT | BILBERRY | CANOPY TYPE | EASTING | MGT | ROAD | SETTLE | | SUCCESSION | TOURI_S | CANOPY COV | | WATER | SLOPE | |  | |  | df | logLik | AIC | | | delta | |
| **2048** | **2.1902** | **0.0224** | **+** | **-0.4089** | **+** | **-0.0005** | **-0.0003** | | **+** | **-0.0009** | **-0.0363** | | **-0.0012** | **-0.0416** | |  | |  | **20** | **-3638.5** | **7317.0** | | | **0.00** | |
| 1024 | 2.0493 | 0.0212 | + | -0.4032 | + | -0.0005 | -0.0002 | | + | -0.0011 | -0.0368 | | NA | -0.0414 | |  | |  | 19 | -3643.8 | 7325.6 | | | 8.63 | |
|  |  |  |  |  |  |  |  | |  |  |  | |  |  | |  | |  |  |  |  | | |  | |
|  | Selection in home range during winter: day | | | | |  |  | |  |  |  | |  |  | |  | |  |  |  |  | | |  | |
| NR | INTERCEPT | EASTING | CANOPY TYPE | MGT | FEED | HUNT | NORTHING | | TOURI_W | TREECOVER |  | |  |  | |  | |  | df | logLik | AIC | | | delta | |
| **256** | **0.4899** | **-0.2306** | **+** | **+** | **-0.0020** | **-0.0010** | **0.2415** | | **0.0006** | **-0.0118** |  | |  |  | |  | |  | **14** | **-2019.5** | **4066.9** | | | **0.00** | |
| 254 | 0.5887 | NA | + | + | -0.0020 | -0.0010 | 0.2378 | | 0.0005 | -0.0114 |  | |  |  | |  | |  | 13 | -2023.5 | 4073.0 | | | 6.10 | |
|  |  |  |  |  |  |  |  | |  |  |  | |  |  | |  | |  |  |  |  | | |  | |
|  | Selection in home range during winter: night | | | | |  |  | |  |  |  | |  |  | |  | |  |  |  |  | | |  | |
| NR | INTERCEPT | EASTING | HUNT | CANOPY COV | MGT | NORTHING | PROTECT_W | | ROAD | TOURI_W | SLOPE | | SUCCESSION | WATER | |  | |  | df | logLik | AIC | | | delta | |
| **2048** | **1.6983** | **-0.9826** | **0.0007** | **-0.0247** | **+** | **-0.2749** | **-0.0131** | | **-0.0018** | **-0.0028** | **-0.0999** | | **+** | **-0.0021** | |  | |  | **17** | **-1746.3** | **3526.7** | | | **0.00** | |
| 2046 | 1.8766 | -0.9638 | NA | -0.0241 | + | -0.2827 | -0.0133 | | -0.0019 | -0.0029 | -0.0936 | | + | -0.0020 | |  | |  | 16 | -1751.2 | 3534.4 | | | 7.74 | |
