# Supplementary material for: Human recreation affects spatio-temporal habitat use patterns in red deer (Cervus elaphus)
Source: PLoS One. 2017 May 3;12(5):e0175134. doi: 10.1371/journal.pone.0175134 (PMC5414982; doi:10.1371/journal.pone.0175134)
Supplement: S5 Table — The table shows the variables included in the five component models (with variable codes described below), as well as the relative importance of the variables. (DOCX) [file pone.0175134.s008.docx]

Supporting Information PONE-D-16-42033R2

**Coppes et al. 2017: Human recreation affects spatio-temporal habitat use patterns in red deer (Cervus elaphus)**

**S5 Table: Model averaging of the model describing home range selection in study area during summer (Table 3a).** The table shows the variables included in the five component models (with variable codes described below), as well as the relative importance of the variables.

| Component models: | |  |  |  |  |  |
| --- | --- | --- | --- | --- | --- | --- |
|  | df | logLik | AIC | delta | weight |  |
| 1/2/3/4/5/6/8/10/12 | 18 | -12087,47 | 24210,95 | 0,00 | 0,31 |  |
| 1/2/3/4/5/6/8/9/10/12 | 19 | -12086,83 | 24211,66 | 0,71 | 0,22 |  |
| 1/2/3/4/5/6/8/9/10/11/12 | 20 | -12086,00 | 24212,00 | 1,05 | 0,18 |  |
| 1/2/3/4/5/6/8/10/11/12 | 19 | -12087,00 | 24212,01 | 1,06 | 0,18 |  |
| 1/2/3/4/5/6/7/8/10/12 | 19 | -12087,47 | 24212,95 | 2,00 | 0,11 |  |
|  |  |  |  |  |  |  |
| Variable | BILBERRY | CANOPY_TYPE | FOREST250 | HERB_GRAS | HUNT | MGT |
| Term code: | 1 | 2 | 3 | 4 | 5 | 6 |
|  | PROTECT_S | SETTLE | SLOPE | SUCCESSION | TOURI_S | WATER |
|  | 7 | 8 | 9 | 10 | 11 | 12 |
|  |  |  |  |  |  |  |
| Relative variable importance: | |  |  |  |  |  |
|  | BILBERRY | CANOPY_TYPE | FOREST250 | HERB_GRAS | HUNT | MGT |
| Importance: | 1 | 1 | 1 | 1 | 1 | 1 |
| N containing models | 5 | 5 | 5 | 5 | 5 | 5 |
|  | SETTLE | SUCCESSION | WATER | SLOPE | TOURI_S | PROTECT_S |
| Importance: | 1 | 1 | 1 | 0,4 | 0,36 | 0,11 |
| N containing models | 5 | 5 | 5 | 2 | 2 | 1 |
