# Supplementary material for: Human recreation affects spatio-temporal habitat use patterns in red deer (Cervus elaphus)
Source: PLoS One. 2017 May 3;12(5):e0175134. doi: 10.1371/journal.pone.0175134 (PMC5414982; doi:10.1371/journal.pone.0175134)
Supplement: S6 Table — For categorical variables the corrected VIF values (VIF^(1/2Df)) are provided. (DOCX) [file pone.0175134.s009.docx]

Supporting Information PONE-D-16-42033R2

**Coppes et al. 2017: Human recreation affects spatio-temporal habitat use patterns in red deer (Cervus elaphus)**

**S6 Table: Variation inflation factors (VIF) for the variables included in the models presented in Table 3 and 4.** For categorical variables the corrected VIF values (VIF^(1/2Df)) are provided.

| Selection of home range in study area summer | | | | | Selection of home range in study area winter | | | |
| --- | --- | --- | --- | --- | --- | --- | --- | --- |
|  | VIF | Df | VIF^(1/2Df) |  |  | VIF | Df | VIF^(1/2Df) |
| SUCCESSION | 87.8297 | 4 | 1.7497 |  | SUCCESSION | 11.5638 | 4 | 1.3580 |
| PROTECT_S | 2.8095 | 1 | 1.6762 |  | PROTECT_W | 1.7148 | 1 | 1.3095 |
| BILBERRY | 1.3562 | 1 | 1.1646 |  | CANOPY_COV | 6.5056 | 1 | 2.5506 |
| CANOPY_TYPE | 76.2731 | 4 | 1.7191 |  | WATER | 1.2278 | 1 | 1.1081 |
| HERB_GRAS | 2.2372 | 1 | 1.4957 |  | EASTING | 1.1985 | 1 | 1.0948 |
| WATER | 1.2261 | 1 | 1.1073 |  | NORTHING | 1.2435 | 1 | 1.1151 |
| FOREST250 | 4.4706 | 1 | 2.1144 |  | MGT | 2.7752 | 2 | 1.2907 |
| SLOPE | 1.7117 | 1 | 1.3083 |  | HUNT | 1.2186 | 1 | 1.1039 |
| MGT | 2.8591 | 2 | 1.3003 |  | SETTLE | 2.1787 | 1 | 1.4760 |
| HUNT_ | 1.2072 | 1 | 1.0987 |  | ROAD | 2.2986 | 1 | 1.5161 |
| SETTLE | 1.6692 | 1 | 1.2920 |  | FEED | 1.9641 | 1 | 1.4015 |
| TOURI_S | 1.7299 | 1 | 1.3153 |  |  |  |  |  |
|  |  |  |  |  |  |  |  |  |
| Selection in home range summer day | | | |  | Selection in home range summer night | | | |
|  | VIF | Df | VIF^(1/2Df) |  |  | VIF | Df | VIF^(1/2Df) |
| SUCCESSION | 26.0145 | 4 | 1.5028 |  | CANOPY_COV | 6.2753 | 1 | 2.5051 |
| CANOPY_COV | 6.0301 | 1 | 2.4556 |  | SUCCESSION | 66.9894 | 4 | 1.6914 |
| TOURI S | 2.2092 | 1 | 1.4864 |  | BILBERRY | 1.3733 | 1 | 1.1719 |
| FOREST250 | 7.5032 | 1 | 2.7392 |  | CANOPY_TYPE | 52.3784 | 4 | 1.6402 |
| MGT | 6.2553 | 2 | 1.5815 |  | WATER | 1.3645 | 1 | 1.1681 |
| WATER | 1.4176 | 1 | 1.1906 |  | EASTING | 1.0917 | 1 | 1.0448 |
| HUNT | 1.3621 | 1 | 1.1671 |  | SLOPE | 1.8101 | 1 | 1.3454 |
| BILBERRY | 1.4868 | 1 | 1.2194 |  | MGT | 3.8503 | 2 | 1.4008 |
| PROTECT_S | 2.5759 | 1 | 1.6050 |  | TOURI_ S | 1.8544 | 1 | 1.3618 |
| UNDER_TYPE | 3.5834 | 4 | 1.1730 |  | ROAD | 2.3282 | 1 | 1.5258 |
| SLOPE | 1.9711 | 1 | 1.4040 |  | SETTLE | 2.6390 | 1 | 1.6245 |
| NORTHING | 1.1644 | 1 | 1.0791 |  |  |  |  |  |
| EASTING | 1.1195 | 1 | 1.0581 |  |  |  |  |  |
|  |  |  |  |  |  |  |  |  |
| Selection in home range winter day | | | |  | Selection in home range winter night | | | |
|  | VIF | Df | VIF^(1/2Df) |  |  | VIF | Df | VIF^(1/2Df) |
| CANOPY_COV | 8.4449 | 1 | 2.9060 |  | SUCCESSION | 4.6354 | 4 | 1.2113 |
| CANOPY_TYPE | 16.0006 | 4 | 1.4142 |  | SUCCESSION | 1.6708 | 1 | 1.2926 |
| NORTHING | 1.3806 | 1 | 1.1750 |  | NORTHING | 1.2164 | 1 | 1.1029 |
| EASTING | 1.0975 | 1 | 1.0476 |  | EASTING | 1.1893 | 1 | 1.0906 |
| MGT | 6.0831 | 2 | 1.5705 |  | SLOPE | 3.2211 | 1 | 1.7947 |
| HUNT | 1.5674 | 1 | 1.2519 |  | WATER | 1.4825 | 1 | 1.2176 |
| TOURI W | 2.0509 | 1 | 1.4321 |  | MGT | 4.9732 | 2 | 1.4933 |
| FEED | 2.6154 | 1 | 1.6172 |  | HUNT | 1.8869 | 1 | 1.3736 |
|  |  |  |  |  | TOURI W | 1.8581 | 1 | 1.3631 |
|  |  |  |  |  | ROAD | 2.7571 | 1 | 1.6605 |
