# Supplementary material for: Human recreation affects spatio-temporal habitat use patterns in red deer (Cervus elaphus)
Source: PLoS One. 2017 May 3;12(5):e0175134. doi: 10.1371/journal.pone.0175134 (PMC5414982; doi:10.1371/journal.pone.0175134)
Supplement: S7 Table — Odd’s ratios were obtained using the Wald chi-square method, the lower (2.5%) and upper (97.5%) boundary of the confidence interval are provided. (DOCX) [file pone.0175134.s010.docx]

Supporting Information PONE-D-16-42033R2

**Coppes et al. 2017: Human recreation affects spatio-temporal habitat use patterns in red deer (Cervus elaphus)**

**S7 Table: Odd’s ratios with 95% confidence interval for the coefficients of the models provided in Table 3 and 4.** Odd’s ratios were obtained using the Wald chi-square method, the lower (2.5%) and upper (97.5%) boundary of the confidence interval are provided.

| Selection of home range in study area summer | | | |  | Selection of home range in study area winter | | | |
| --- | --- | --- | --- | --- | --- | --- | --- | --- |
|  | Estimate | 2.5% | 97.5% |  |  | Estimate | 2.5% | 97.5% |
| INTERCEPT | 0.1578 | 0.1237 | 0.2013 |  | INTERCEPT | 19.6242 | 13.6476 | 28.2182 |
| SUC_REGTHICK | 1.5718 | 0.9098 | 2.7154 |  | SUC_REGTHICK | 9.1042 | 5.6333 | 14.7135 |
| SUC_POLE | 0.9222 | 0.5420 | 1.5689 |  | SUC_POLE | 2.7893 | 1.8467 | 4.2131 |
| SUC_TREE | 1.0318 | 0.6088 | 1.7486 |  | SUC_TREE | 5.8740 | 4.0445 | 8.5312 |
| SUC_OLD | 0.8230 | 0.4812 | 1.4077 |  | SUC_OLD | 6.6752 | 4.5287 | 9.8392 |
| PROTECT_S | 0.9999 | 0.9981 | 1.0018 |  | CANOPY_COVER | 0.9895 | 0.9847 | 0.9943 |
| BILBERRY | 0.9916 | 0.9881 | 0.9950 |  | PROTECT_W | 0.9901 | 0.9863 | 0.9939 |
| CAN_CON | 0.3773 | 0.2222 | 0.6407 |  | EASTING | 0.3486 | 0.3137 | 0.3874 |
| CAN_DEC | 1.4863 | 0.6585 | 3.3549 |  | NORTHING | 0.7636 | 0.6975 | 0.8360 |
| CAN_CONMIX | 0.4213 | 0.2479 | 0.7158 |  | WATER | 1.0009 | 1.0003 | 1.0014 |
| CAN_DECMIX | 0.4763 | 0.2765 | 0.8206 |  | MGT_CORE | 0.6076 | 0.5093 | 0.7248 |
| HERB_GRAS | 1.0177 | 1.0162 | 1.0193 |  | MGT_REFUGE | 1.6971 | 1.3290 | 2.1671 |
| WATER | 0.9989 | 0.9985 | 0.9992 |  | HUNT | 0.9981 | 0.9979 | 0.9983 |
| FOREST250 | 1.7508 | 1.4181 | 2.1616 |  | FEED | 0.9980 | 0.9978 | 0.9981 |
| SLOPE | 0.9958 | 0.9899 | 1.0017 |  | ROAD | 0.9993 | 0.9991 | 0.9995 |
| MGT_CORE | 6.8521 | 6.0446 | 7.7676 |  | SETTLE | 0.9996 | 0.9995 | 0.9998 |
| MGT_REFUGE | 15.2986 | 13.1606 | 17.7839 |  |  |  |  |  |
| HUNT | 0.9982 | 0.9981 | 0.9983 |  |  |  |  |  |
| SETTLE | 1.0004 | 1.0003 | 1.0004 |  |  |  |  |  |
| TOURI_S | 0.9998 | 0.9996 | 1.0001 |  |  |  |  |  |
|  |  |  |  |  |  |  |  |  |
| Selection in home range, summer during day | | | |  | Selection in home range, summer during night | | | |
|  | Estimate | 2.5% | 97.5% |  |  | Estimate | 2.5% | 97.5% |
| INTERCEPT | 0.0093 | 0.0060 | 0.0145 |  | INTERCEPT | 8.9371 | 6.1799 | 12.9244 |
| CANOPY_COVER | 0.9873 | 0.9834 | 0.9913 |  | CANOPY_COVER | 0.9644 | 0.9600 | 0.9688 |
| SUC_REGTHICK | 11.1557 | 7.1873 | 17.3152 |  | SUC_REGTHICK | 1.6449 | 0.6596 | 4.1019 |
| SUC_POLE | 3.8622 | 2.5874 | 5.7653 |  | SUC_POLE | 2.1013 | 0.8920 | 4.9500 |
| SUC_TREE | 1.9211 | 1.3011 | 2.8366 |  | SUC_TREE | 2.0798 | 0.8904 | 4.8579 |
| SUC_OLD | 2.6691 | 1.7945 | 3.9701 |  | SUC_OLD | 1.2245 | 0.5209 | 2.8785 |
| BILBERRY | 0.9757 | 0.9695 | 0.9819 |  | BILBERRY | 1.0226 | 1.0162 | 1.0291 |
| PROTECT_S | 0.9960 | 0.9933 | 0.9987 |  | CAN_CON | 0.4675 | 0.2002 | 1.0915 |
| UNDER_CON | 2.0772 | 1.7065 | 2.5286 |  | CAN_DEC | 1.4997 | 0.4474 | 5.0276 |
| UNDER_DEC | 6.3580 | 3.9313 | 10.2827 |  | CAN_CONMIX | 0.5076 | 0.2170 | 1.1876 |
| UNDER_CONMIX | 1.9613 | 1.6795 | 2.2903 |  | CAN_DECMIX | 0.2646 | 0.1057 | 0.6621 |
| UNDER_DECMIX | 1.1589 | 0.9755 | 1.3767 |  | WATER_DIST | 0.9988 | 0.9981 | 0.9995 |
| WATER | 1.0025 | 1.0020 | 1.0030 |  | EASTING | 0.6644 | 0.5965 | 0.7400 |
| EASTING | 1.0869 | 1.0018 | 1.1792 |  | SLOPE | 0.9592 | 0.9479 | 0.9708 |
| SLOPE | 1.0658 | 1.0566 | 1.0750 |  | MGT_CORE | 3.9398 | 3.0171 | 5.1446 |
| NORTHING | 0.7568 | 0.7060 | 0.8112 |  | MGT_REFUGE | 5.6906 | 4.1367 | 7.8284 |
| FOREST250 | 4.8705 | 3.1412 | 7.5518 |  | TOURI_S | 0.9991 | 0.9987 | 0.9996 |
| MGT_CORE | 3.6041 | 2.6455 | 4.9101 |  | SETTLE | 0.9998 | 0.9996 | 0.9999 |
| MGT_REFUGE | 3.4858 | 2.5208 | 4.8200 |  | ROAD | 0.9995 | 0.9993 | 0.9997 |
| TOURI_S | 1.0016 | 1.0012 | 1.0020 |  |  |  |  |  |
| HUNT | 0.9988 | 0.9986 | 0.9991 |  |  |  |  |  |
|  |  |  |  |  |  |  |  |  |
| Selection in home range, winter during day | | | |  | Selection in home range, winter during night | | | |
|  | Estimate | 2.5% | 97.5% |  |  | Estimate | 2.5% | 97.5% |
| INTERCEPT | 1.6350 | 0.8231 | 3.2480 |  | INTERCEPT | 6,8880 | 4,4850 | 11,9700 |
| CANOPY_COVER | 0.9883 | 0.9810 | 0.9960 |  | SUC_REGTHICK | 0,9760 | 0,4360 | 1,7690 |
| CAN_CON | 43.0125 | 20.4603 | 90.4220 |  | SUC_POLE | 5,7430 | 0,2030 | 0,4710 |
| CAN_DEC | 17.9391 | 1.2019 | 267.7520 |  | SUC_TREE | 1,7480 | 1,8960 | 3,5260 |
| CAN_CONMIX | 30.2577 | 14.1850 | 64.5420 |  | SUC_OLD | 12,9440 | 2,4150 | 5,7010 |
| CAN_DECMIX | 41.2635 | 16.9385 | 100.5210 |  | PROTECT_W | 16,6010 | 0,9870 | 1,0010 |
| NORTHING | 1.2730 | 1.1013 | 1.4720 |  | NORTHING | 0,9880 | 0,6300 | 0,8630 |
| EASTING | 0.7940 | 0.6771 | 0.9310 |  | EASTING | 0,7390 | 0,2870 | 0,4240 |
| MGT_CORE | 0.0876 | 0.0611 | 0.1260 |  | SLOPE_MEAN | 0,3550 | 0,8780 | 0,9130 |
| MGT_REFUGE | 0.2281 | 0.1454 | 0.3580 |  | WATER | 0,9010 | 0,9960 | 0,9980 |
| HUNT | 0.9990 | 0.9985 | 0.9990 |  | MGT_CORE | 0,9980 | 0,4850 | 0,9170 |
| TOURI_W | 1.0006 | 1.0002 | 1.0010 |  | MGT_REFUGE | 0,7280 | 3,8220 | 8,1110 |
| FEED | 0.9980 | 0.9978 | 0.9980 |  | HUNT | 4,8310 | 1,0000 | 1,0010 |
|  |  |  |  |  | TOURI_W | 1,0010 | 0,9970 | 0,9980 |
|  |  |  |  |  | ROAD | 0,9970 | 0,9980 | 0,9980 |
